# Thermo/pH Responsive Star and Linear Copolymers Containing a Cholic Acid-Derived Monomer, *N*-Isopropylacrylamide and Acrylic Acid: Synthesis and Solution Properties

**DOI:** 10.3390/polym11111859

**Published:** 2019-11-11

**Authors:** Ana Castro-Hernández, Norma Aidé Cortez-Lemus

**Affiliations:** Centro de Graduados e Investigación en Química, Tecnológico Nacional de México/Instituto Tecnológico de Tijuana, A. P. 1166. Tijuana C.P. 22000, B. C., Mexico; analichdez09@gmail.com

**Keywords:** Block copolymer, RAFT, aggregates, cholic acid, PNIPAM, star

## Abstract

In this work three CTAs trithiocarbonate-type were synthesized—bifunctional (with PEG), trifunctional (with glycerol), and tetrafunctional (PERT)—and used in the controlled polymerization of 2-(acryloyloxy)ethyl cholate (CAE) via reversible addition-fragmentation chain transfer (RAFT) polymerization. The resulting macroCTAs containing a cholic acid-derived polymer were chain extended with *N*-isopropylacrylamide with or without acrylic acid. The thermosensitive and/or pH properties of these copolymers were studied in PBS solutions. The copolymers synthesized without poly(acrylic acid) (PAAc) were unstable above the transition temperature. Similar behavior was observed for the copolymer solutions containing PAAc (2% in feed) at lower values of pH showing a faster precipitation above the LCST. On the contrary, copolymer solutions containing PAAc showed great stability at higher pH values for a longer time period at 37 °C. Interestingly, the *D_h_* of the aggregates ranged from 18 to 30 nm in all copolymers (with or without PAAc) below the transition temperature, although the topology and the block sequence in the chain were significantly different.

## 1. Introduction

Cholic acid is a biocompatible, amphiphilic and multifunctional molecule that has been used in several fields such as biomedical, pharmacological, polymer science, and others, due to the versatility of the possibility of chemical modifications [1,2]. Polymers based on cholic acid are attractive in systems designed for drug delivery, performing as an efficient reservoir of hydrophobic molecules. In a pioneering work, Zhu and co-workers designed, synthesized and studied extensively monomers and polymers derived from cholic acid [3,4,5,6,7]. For example, they prepared water-soluble *N*-isopropylacrylamide and (meth)acrylamide copolymers with cholic acid pendant groups [5]. Recently, they prepared block copolymers containing glucosamine and cholic acid via RAFT. Stable aggregates (in the range of 50 from 70 nm) in aqueous solution were prepared and used to demonstrate its capacity as reservoirs of hydrophobic molecules [8]. The multifunctionality of the cholic acid, was also used to prepare star polymers with three and four arms in which the –OH and –COOH groups act as initiating sites of the arms [9,10].

Poly(*N*-isopropylacrylamide) (PNIPAM) is a thermosensitive polymer with an LCST (lower critical solution temperature) approximately at 32 °C [11]. Below this temperature, the amide group interacts with water molecules and acts as hydrogen donor (–NH–) or by accepting hydrogen (C=O) [12]. When PNIPAM is copolymerized with pH responsive or hydrophobic polymers, its transition temperature can be strongly affected. Poly(acrylic acid) (PAAc) is a pH responsive polymer that can alter the transition temperature of PNIPAM [13,14]. In a poly(*N*-isopropylacrylamide)-*b*-poly(acrylic acid) system (maintaining low the molar ratio of PAAc), at pH < 4.25, total protonation of the carboxylate group is observed, enhancing the formation of hydrogen bonding with the amide group of PNIPAM displacing water molecules [15,16]. This interaction leads to a decrease in the LCST of PNIPAM and in many cases, the copolymer becomes water insoluble. On the contrary, at pH 7.4 the carboxyl groups in PAAc are ionized and contributed to the increase of the LCST of PNIPAM [17].

Precise synthesis of star polymers by RAFT has become increasingly important in recent years [18]. The synthesis of star-like polymers by the core-first method via RAFT polymerization, allows polymers to be obtained having the Z-group linked to the core (Z-RAFT approach) or the leaving R-group attached to the core (R-RAFT approach). As an example, star polymers from acrylates (methyl acrylate, butyl acrylate) [19,20], styrene [20], and NIPAM [11], have been generated using the Z-RAFT approach. Moreover, R-RAFT star polymerization of acrylamides (NIPAM and *N*,*N*-dimethylacrylamide) [21,22,23], was found to be efficient in producing star polymers with narrow and monomodal molecular weight distribution. It is important to mention that the multifunctional trithiocarbonate-type was selected as the RAFT agent in the mentioned polymerizations. However, to the best of our knowledge, no study has reported on star polymers based on a cholic acid-derived monomer using R-RAFT polymerization. Some papers reported the synthesis of star polymers using the cholic acid only as a multifunctional initiating core [9,10]. In fact, we believe that there are only a few reports on RAFT polymerization using cholic acid-derived monomer [8,24].

Literature abounds with reports devoted to the development of highly stable micellar aggregates from amphiphilic block copolymers and their applications as nanocarriers for drug delivery [25,26,27,28,29,30]. Several strategies to improve micellar stability of the carriers include end-functionalization with a hydrophilic polymer (PEG or glycopolymers) [31,32]. Also, the design and synthesis of new amphiphilic block copolymers having different topology has been part of research strategies [33,34]. In many cases, papers report drug delivery studies using large sizes of carriers (or aggregates), however, it is important first to demonstrate micellar stability.

In this present work were synthesized three chain transfer agents (CTAs) trithiocarbonate-type—bifunctional with PEG (2000 g/mol), trifunctional (with glycerol ethoxylate ~1000 g/mol) and tetrafunctional (PERT)—and used in the polymerization of cholic acid-derived monomer via reversible addition-fragmentation chain transfer (RAFT) polymerization. The multifunctional CTAs were designed to generated polymers in which the SC=SS group is located at the polymer chain end. The resulting macroCTAs containing cholic acid-derived polymer (PCAE) were chain extended with NIPAM and NIPAM/AAc. The thermosensitive and pH properties of these copolymers were studied in buffer. Moreover, the stability of the copolymer samples was followed by measuring the *D_h_* (hydrodynamic diameter) using dynamic light scattering (DLS). Below the LCST, the aggregates revealed similar *D_h_*, although the self-assembly takes place from linear as well as star architectures, also, the block sequence was significantly different in the copolymers.

**Scheme 1:**
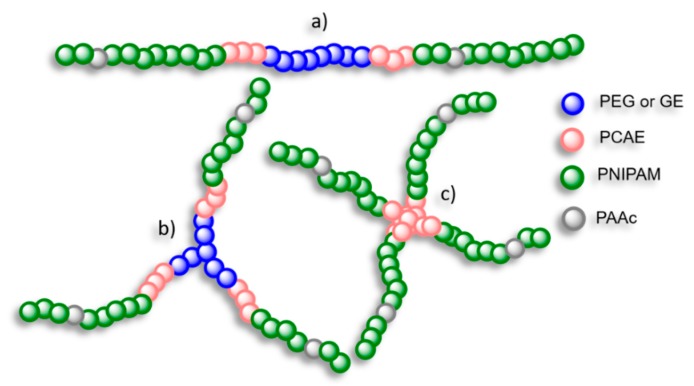
Representation of copolymers synthesized with acrylic acid: (**a**) PAAc-*co*-PNIPAM-*b*-PCAE-*b*-PEG-*b*-PCAE-*b*-PNIPAM-*co*-PAAc), (**b**) (GE-*b*-PCAE-*b*-PNIPAM-*co*-PAAc)_3_, (**c**) (PCAE-*b*-PNIPAM-*co*-PAAc)_4_.

Scheme 1 shows a representation of the copolymers synthesized with acrylic acid having a different topology and block sequence of the polymer segments:

## 2. Experimental

### 2.1. Materials

The materials used were as follows: Penta-erythritol, (PERT, 99%, Aldrich), poly(ethylene glycol) (2000 g/mol, Aldrich, Toluca, Mexico), glycerol ethoxylate (~1000 g/mol, Aldrich), triethylamine (TEA, 99.5%), pyridine (99.8%, Sigma Aldrich, Toluca, Mexico), 2-bromopropionyl bromide (Aldrich, 97%), 4-(dimethylamino) pyridine (95%, Aldrich), carbon disulfide (99.9%, Sigma Aldrich), 1-dodecanethiol (98%, Sigma Aldrich), 2,2′-azobis(2-methylpropionitrile) (98%, Aldrich), cholic acid (98%, Sigma Aldrich), acrylic acid (99%, Aldrich), *N*-isopropylacrylamide (97%, Sigma Aldrich), acetic anhydride (99%, Alfa Aesar), *N*,*N*′-dicyclohexylcarbodiimide (99%, Sigma Aldrich), 2-hydroxyethyl acrylate (96%, Sigma Aldrich), sodium bicarbonate (NaHCO_3_) (100%, Fermont, Monterrey, Mexico), anhydrous sodium sulphate (Na_2_SO_4_), *N*,*N*-dimethylformamide (DMF, Fermont, 99.8%), tetrahydrofuran (THF, Fermont, 99.9%), hydrochloric acid (HCl, 10% aqueous solution, Fermont). Column chromatographic purifications were performed using silica gel (70–230 mesh, Acros Organics).

### 2.2. Characterization

*Nuclear magnetic resonance* (^1^H and ^13^C NMR) spectra were collected at 400 MHz or 100 MHz on a Bruker AMX-400 spectrometer at 298 K. Chemical shifts are reported as δ in parts per million (ppm) using TMS as internal standard. NMR spectra were processed using MestReNova NMR software. The solvent used was deuterated chloroform (CDCl_3_) and deuterated methanol (CD_3_OD) for samples. Data is reported as follows: chemical shift—multiplicity (*s* = singlet, *d* = doublet, *dd* = doublet of doublets, *t* = triplet, *t*_d_ = triplet of doublets, *q* = quartet, *q* = quintet, *m* = multiplet). Dynamic light scattering (DLS) measurements were carried out at different concentrations in PBS (0.5 to 7.0 mg/mL) using a Malvern Instruments Nano-ZS Nanosizer (ZEN 3690) equipment. The instrument is equipped with a helium neon laser (633 nm) with a size detection range of 0.6 nm–5 μm. DLS experiments were performed at the scattering angle of 90° and equilibrated for 2 min before data collection. The solutions/dispersions were filtered through a 0.45 μm nylon membrane filter before analysis to remove dust. The volume-average hydrodynamic diameter (*D_h_*) and polydispersity index (PDI) were calculated using Malvern Instruments dispersion technology software, based on CONTIN analysis and the Stokes–Einstein equation for spheres as usual. The LCST was taken as the temperature at which the copolymer was still soluble (before the solution started to turn cloudy). The LCST was measured by DLS using the Nano-ZS Nanosizer equipment with a temperature program that increased from 25 °C to 50 °C in two degree steps, equilibrating for 2 min once the measurement temperature was achieved; measurements were performed three times, each of which included three 30 s runs. Gel permeation chromatography (GPC) was performed on a Varian 9002 chromatograph equipped with a series of three columns (Phenogel: OH-646-K0, OH-645-K0 and OH-643-K0) and two detectors: a refractive index detector (Varian RI-4) and a triangle light scattering detector (LS detector MINI-DAWN, Wyatt). The measurements were performed in THF at 35 °C. Polystyrene standards were used for calibration of the LS detector. THF was used as the mobile phase at a flow rate of 0.7 mL/min. Sample solutions were prepared using 20 mg/mL concentration and filtered through a 0.45 μm PTFE membrane filter before analysis.

## 3. Results and Discussion

The CTAs trithiocarbonate-type were designed to polymerize 2-(acryloyloxy)ethyl cholate (cholic acid-derived monomer, CAE) by RAFT. CAE monomer was synthesized following the methodology of De and co-workers [24]. The ^1^H NMR spectrum of cholic acid-derived monomer is displayed in Figure S1 (Supporting information file).

### 3.1. Synthesis of the CTAs 1 to 3

**Scheme 2:**
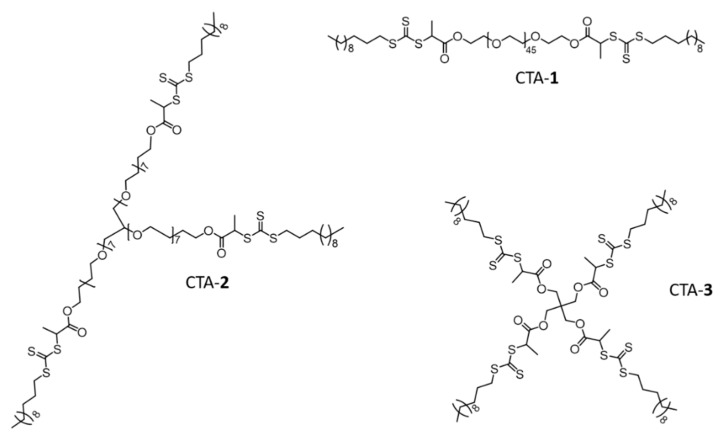
Structure of CTAs: bifunctional CTA-**1** (core of PEG_2000 g/mol_), trifunctional CTA-2 (core of glycerol_1000 g/mol_) and tetrafunctional CTA-3 (core of penta-erythritol).

Bifunctional, trifunctional and tetrafunctional CTAs 1 to 3 (Scheme 2) were easily synthesized by the addition of CS_2_ to dodecanethiol in the presence of triethylamine; later, the corresponding brominated precursor was added to the reaction. The brominate precursors were synthesized by the reaction of 2-bromopropionyl bromide with molecules or macromolecules that contain hydroxyl groups: PEG (2000 g/mol, two hydroxyl groups), glycerol ethoxylate (1000 g/mol, three hydroxyl groups) and PERT (four hydroxyl groups). In the ^1^H NMR spectrum of CTA-1-Br precursor (Figure S2) four distinctive peaks were observed: a quartet peak at 4.43 to 4.36 ppm attributed to the methine “a”; a triplet peak “b” was observed at 4.32 ppm attributed to the methylene of PEG; the integration value of 4 confirmed the complete functionalization of the hydroxyl end group of PEG; the peak “d” that corresponded to the methylene groups of the repetitive unit of PEG was observed at 3.66 ppm; a doublet peak “e” at 1.82 ppm corresponded to the hydrogens of the two methyl groups adjacent to the bromide. In the ^13^C NMR spectrum of CTA-1-Br precursor (Figure S3) a distinctive peak “a” at 170.2 ppm was assigned to the C=O group. In the ^1^H NMR spectrum of CTA-1 (Figure S4), a quartet peak at 4.88 to 4.79 ppm was attributed to the methine “a”; a triplet peak “b” was observed at 4.29 ppm attributed to the methylene of PEG; the peak “d” that corresponded to the methylene of the repetitive unit of PEG was observed at 3.66 ppm; a doublet peak “g” at 1.60 ppm was assigned to the two methyl groups close to the trithiocarbonate group; the triplet peak “j” at 0.88 ppm corresponded to the end methyl group of the dodecyl chain. In the ^13^C NMR spectrum a peak “a” at 221.9 and the peak “b” at 171 ppm corresponded to C=S and C=O groups, respectively (Figure S5).

The synthesis of CTA-2-Br precursor starts from trifunctional glycerol ethoxylate (1000 g/mol). The ^1^H and ^13^C NMR spectrum for the CTA-2-Br precursor is displayed in Figures S6 and S7, respectively. The integral values of peaks “a” and “b” are three and six, respectively, and demonstrated the complete functionalization of glycerol ethoxylate. The peak “e” assigned for the methyl group was observed at 1.82 ppm. The peak “a” corresponded to the C=O group was observed at 170.2 ppm (Figure S7). The complete functionalization of the CTA-2 was confirmed with the ^1^H NMR displayed in Figure S8. The integral ratio of three between peak “j” (–CH_3_ of the aliphatic chain at 0.9 ppm) and peak “a” displayed at 4.85 ppm confirmed the star-like architecture with three arms. In the ^13^C NMR spectrum (Figure S9) the peak “a” at 221.9 and the peak “b” at 171.1 ppm confirmed the presence of C=S and C=O groups.

The four hydroxyl groups of PERT were reacted with 2-bromopropionyl bromide to obtain the CTA-3-Br precursor. The ^1^H and ^13^C NMR spectrum is displayed in Figures S10 and S11, respectively. A multiple peak (overlapped signal) at 4.32 to 4.22 corresponded to the methylene “a” and methine “b” groups of the molecule. This multiple peak results from a quartet from “a” and four doublets from “b”. The peak “c” was assigned to the methyl group at 1.76 ppm. The integral value of 12 in both peaks confirmed the complete functionalization. In the ^1^H NMR spectrum for CTA-3 (Figure S12), both, the integral value of the methylene “b” (located in the core of the molecule) and the methyl group “h” (end of the aliphatic chain) was 3 and confirmed the tetra functionality of CTA-3. The peak at 221.7 ppm in the ^13^C NMR spectrum confirmed the presence of the C=S group (Figure S13).

### 3.2. Synthesis and Characterization of MacroCTAs 1 to 3 (Also, Polymerization of CAE in the Presence of CTAs)

The CTAs previously synthesized were used to polymerize cholic acid-derived monomer (CAE) by RAFT. CAE monomer was synthesized following the methodology of De and co-workers [24]. Our target was to obtain macroCTAs with comparable content of PCAE (Table 1). The NMR analyses for macroCTAs show the peak characteristics of a cholate skeleton in all cases. The ^1^H NMR spectrum of macroCTA-**1** or PCAE_3_-*b*-PEG_45_-*b*-PCAE_3_ (Figure S14) is described in detail. The peak at 4.89 ppm “f” is assigned to the methine hydrogen (–CHCH_3_) that come from CTA-1. The peaks at 3.99 and 3.85 ppm belong to hydrogens 12′ and 7′ respectively from the cholate skeleton. The overlapped peaks “a, b” at 4.29 ppm corresponded to the methylene hydrogens (–O–CH_2_–CH_2_–O–). The peaks 18′, 21′ and 19′ were assigned to the methyl groups in cholate skeleton. The peak “c” corresponded to the methylene hydrogens (–CH_2_–CH_2_–O–) from PEG. Both, the degree of polymerization (DP) of PCAE as well as the molecular weight by ^1^H NMR of PCAE_3_-*b*-PEG_45_-*b*-PCAE_3_ were calculated comparing the integral value of the “f” peak (RAFT residue) with the integrals value of peak “18′” (Figure S14). By considering the *Mn* of the monomer (506.68 g/mol), DP of PEG and RAFT residue, the molecular weight of macroCTA-1 by ^1^H NMR was 5502 g/mol.

The ^1^H NMR spectrum of (GE_7_-*b*-PCAE_4_)_3_ or macroCTA-2 is presented in Figure S15. The molecular weight by ^1^H NMR of macroCTA-2 was calculated comparing the integral value of the peaks “f”, “18′” and “c”. The molecular weight by ^1^H NMR of macroCTA-2 was 7333 g/mol. The ^1^H NMR spectrum of (PCAE_2_)_4_ or macroCTA-3 is presented in Figure S16. The peaks “b, c” at 4.43–4.03 ppm corresponded to the methylene hydrogens (–O–CH_2_–CH_2_–O–) and overlapped with methylene “a” from the CTA. The molecular weight of macroCTA-3 by ^1^H NMR was calculated comparing the integral value of the “f” peak with the integrals value of peak “18” (Figure S16). Thus, the molecular weight by ^1^H NMR of macroCTA-3 was 5079 g/mol.

**Scheme 3:**
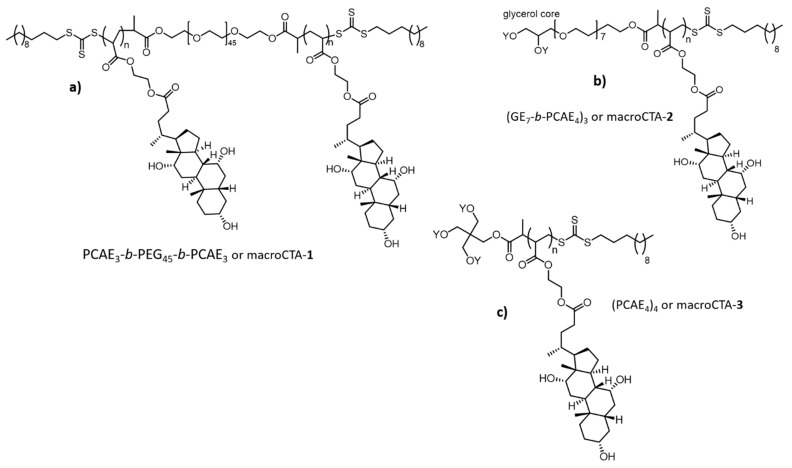
Structure of the macroCTAs: bifunctional macroCTA-1, trifunctional macroCTA-2 and tetrafunctional macroCTA-3.

**Figure 1:**
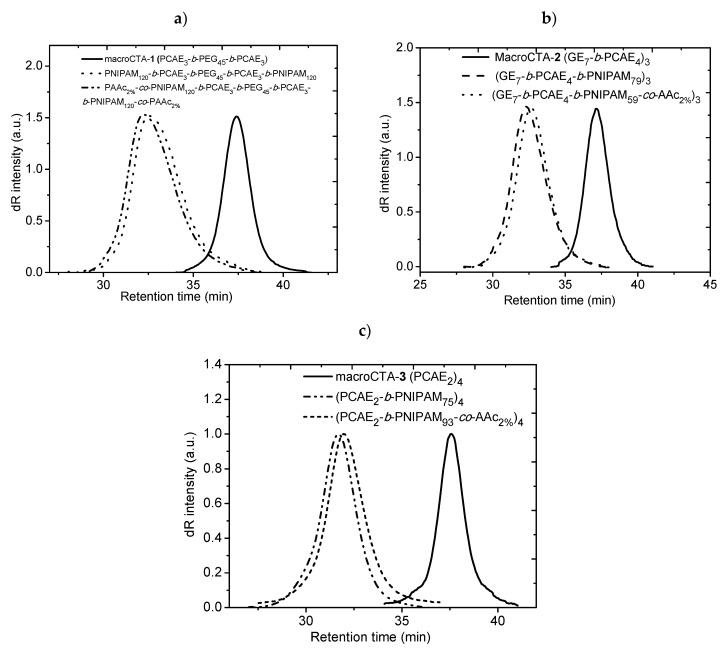
Normalized GPC traces (refractive index) of acetylated samples in THF (**a**) copolymers derived from macroCTA-1 or (PCAE_3_-*b*-PEG_45_-*b*-PCAE_3_); (**b**) copolymers derived from macroCTA-2 or (GE_7_-*b*-PCAE_4_)_3_; (**c**) copolymers derived from macroCTA-3 or (PCAE_2_)_4_.

The PCAE block is the hydrophobic segment in these copolymers and its presence affects dramatically the transition temperature of the PNIPAM chains even in low molar ratio [4,9]. For this reason, PCAE polymers with low molecular weight were synthesized. The chemical structure, the topology, and the block sequence of polymers is displayed in Scheme 3. The polymerization of CAE was carried out in DMF at 68 °C using AIBN as an initiator. Table 1 displays the results of the RAFT polymerization of CAE in the presence of the CTAs denoted as 1 to 3. As can be observed from Figure 1a–c), the GPC traces for the acetylated PCAE polymers derived from CTAs 1 to 3 showed a unimodal molecular weight distribution with acceptable dispersity values (1.06–1.21). The monomer conversion and molecular weights of the macroCTAs are summarized in Table 1. Unfortunately, the accuracy of the d*n*/d*c* values used in the molecular weight calculations were not determined for the macroCTAs and copolymers. The d*n*/d*c* value reported for linear PNIPAM in THF was used in the calculations of the molecular weight, since the PNIPAM content in the copolymers reached more than 75%. As can be observed from Table 1, the theorical molecular weight values were similar to the calculated ones.

### 3.3. Chain Extension Polymerization of the MacroCTAs Containing PCAE with NIPAM or NIPAM/AAc

**Scheme 4:**
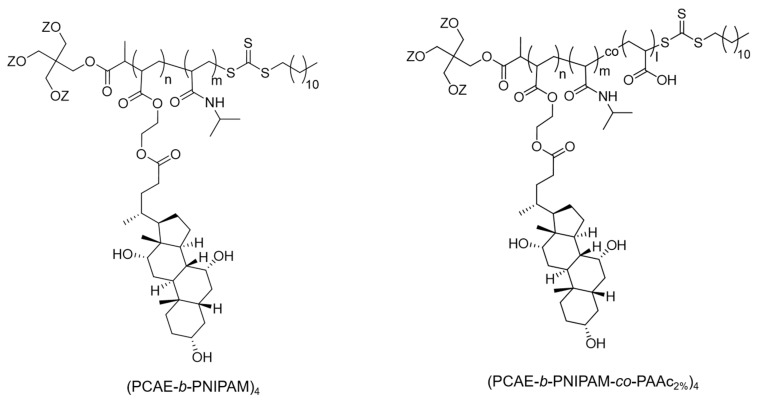
Representative (PCAE_2_-*b*-PNIPAM_75_)_4_ and (PCAE_2_-*b*-PNIPAM_93_-*co*-PAAc_2%_)_4_ copolymers using (PCAE_2_)_4_ or macroCTA-3 as precursor.

The resulting macro-CTAs containing PCAE, were chain extended with *N*-isopropylacrylamide and *N*-isopropylacrylamide/acrylic acid. The polymerization was carried out in DMF at 68 °C using AIBN as an initiator. Table 2 displays the results of the RAFT polymerization of NIPAM and NIPAM-*co*-AAc in the presence of three different macroCTAs (See Scheme 4).

The ^1^H NMR spectra for the samples PNIPAM_120_-*b*-PCAE_3_-*b*-PEG_45_-*b*-PCAE_3_-*b*-PNIPAM_120_ and PAAc_2%_-*co*-PNIPAM_147_-*b*-PCAE_3_-*b*-PEG_45_-*b*-PCAE_3_-*b*-PNIPAM_147_-*co*-AAc_2%_) (Table 2) are shown in Figures S17 and S18, respectively. The characteristics peaks corresponding to PNIPAM (7.24–5.75 ppm, “a”), PEG (3.6 ppm, “b”) and PCAE (0.7 ppm, 18′) are clearly observed. The DP of PNIPAM and PCAE was calculated considering a DP for PEG of 45 (182 hydrogens). The ^1^H NMR spectra for the samples (GE_7_-*b*-PCAE_4_-*b*-PNIPAM_79_)_3_ and (GE_7_-*b*-PCAE_4_-*b*-PNIPAM_59_-*co*-PAAc_2%_)_3_ are shown in Figures S19 and S20. The peak at 3.6 ppm (80 hydrogens) was used to estimate the DP of PNIPAM and PCAE.

The ^1^H NMR spectra for the samples (PCAE_2_-*b*-PNIPAM_75_)_4_ and (PCAE_2_-*b*-PNIPAM_93_-*co*-AAc_2%_)_4_ (Table 2) are shown in Figures S21 and S22, respectively. The DP in these copolymers was estimated considering the DP of PCAE from the macroCTA-3 (estimated by ^1^H NMR) with also the *M*_n_ by GPC of the copolymer. Unfortunately, the estimation of the molecular weight by ^1^H NMR was not possible for these copolymers.

The *M*_n_ value of the copolymers determined by ^1^H NMR is higher than the value obtained by GPC (Table 2). As we mention above, the *M*_n_ of the copolymers analyzed by GPC is inaccurate, because the d*n*/d*c* value was not calculated. In Figure 1 the GPC traces for the acetylated samples of copolymers shifted to shorter retention time with respect to the parent macroCTA demonstrating successful chain extension. Moreover, the GPC curves showed unimodal distribution with acceptable dispersity values (1.28–1.4).

### 3.4. Aqueous Solution Properties of Block Copolymers

DLS was used to determinate the LCST of the copolymer solutions in phosphate buffer saline (PBS) by monitoring the changes in the hydrodynamic diameter upon heating process.

#### 3.4.1. Thermosensitivity Behavior of Block Copolymers Synthesized without Acrylic Acid

**Figure 2:**
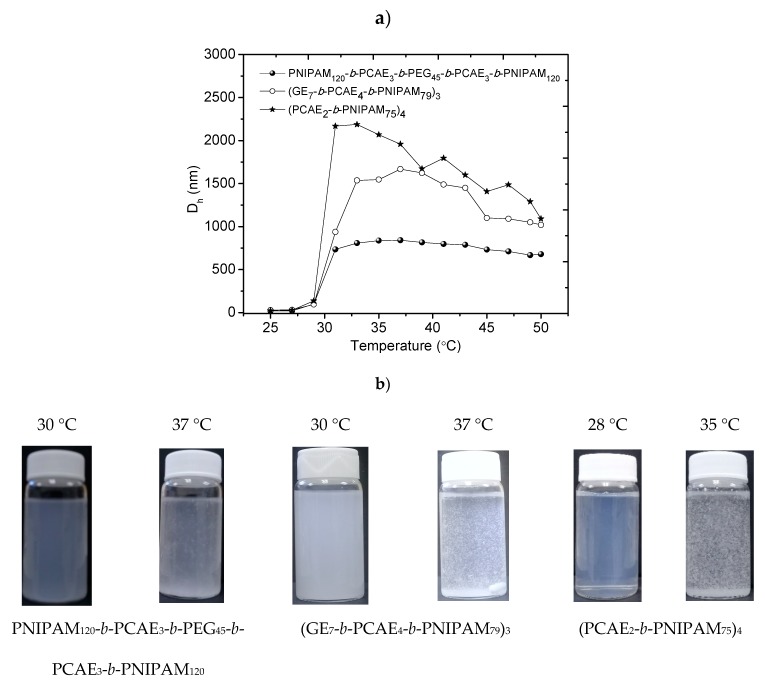
(**a**) DLS measurements of copolymers (*c* = 0.5 mg/mL in PBS). Evolution of the *D_h_* of the copolymers as a function of the temperature. (**b**) Representative image of PNIPAM_120_-*b*-PCAE_3_-*b*-PEG_45_-*b*-PCAE_3_-*b*-PNIPAM_120_, (GE_7_-*b*-PCAE_4_-*b*-PNIPAM_79_)_3_ and (PCAE_2_-*b*-PNIPAM_75_)_4_ copolymers taken at different temperatures.

The amphiphilic copolymers PNIPAM_120_-*b*-PCAE_3_-*b*-PEG_45_-*b*-PCAE_3_-*b*-PNIPAM_120_, (GE_7_-*b*-PCAE_4_-*b*-PNIPAM_79_)_3_ and (PCAE_2_-*b*-PNIPAM_75_)_4_ were synthesized with low content of PCAE (2.2% to 4.4%) (Table 2). From DLS measurements (Figure 2) the copolymers showed an LCST of approximately 29 °C. The PCAE block not only reduced the LCST of PNIPAM but also affected the dispersibility of the copolymers in water or PBS. For instance, the sample PNIPAM_87_-*b*-PCAE_5_-*b*-PEG_45_-*b*-PCAE_5_-*b*-PNIPAM_87_ (Table 2) did not disperse in PBS due to the increase in the PCAE content (4.4%) and to decrease of PNIPAM (76%) compared with the copolymer PNIPAM_120_-*b*-PCAE_3_-*b*-PEG_45_-*b*-PCAE_3_-*b*-PNIPAM_120_ in which PCAE and PNIPAM content was 2% and 82% respectively. Interestingly, at 25 °C, regardless of the macroCTA precursor, the copolymers formed “aggregates” with a *D_h_* ranged from 19 to 25 nm (0.5 mg/mL, in PBS). These samples were also analyzed in THF (0.5 mg/mL) by DLS revealing a *D_h_* of ~8–10 nm (by intensity). On consideration, the self-assembly of two or three copolymeric chains could probably form aggregates with a tight PCAE core. For instance, the self-assembled behavior of the copolymer (GE_7_-*b*-PCAE_4_-*b*-PNIPAM_79_)_3_ was studied at different polymer concentrations. From the DLS plot (Figure S23), the *D_h_* increased from 20 to 45 nm at a concentration of 0.5 to 8 mg/mL respectively (in PBS). The copolymer solution with 7 mg/mL resulted in a transparent appearance, showing good stability only below the LCST. On the other hand, it is important to note that above the LCST, the size of the aggregates decreased for the copolymers containing PEG or GE (see Figure 2). For example, at 31 °C, the *D_h_* was 735, 938, and 2168 nm for the copolymers PNIPAM_120_-*b*-PCAE_3_-*b*-PEG_45_-*b*-PCAE_3_-*b*-PNIPAM_120_, (GE_7_-*b*-PCAE_4_-*b*-PNIPAM_79_)_3_ and (PCAE_2_-*b*-PNIPAM_75_)_4_, respectively.

From DLS measurements displayed in Figure 2, a dramatic increase in the *D_h_* suggest the formation of very large aggregates; this phenomenon has been well studied for PNIPAM polymers as a consequence of the faster dehydration process ascribed to water molecules surrounded isopropyl and methylene groups; this step is followed by the formation of hydrogen bonds intra- and interchain between the amide groups [13,14]. Moreover, at 35 °C these aggregates undergo shrinkage upon heating and a decrease in the *D_h_* was observed. According to the literature, a gradual decrease in the size of micellar aggregates during the heating process corresponds to the formation of smaller micelles [34].

A visual inspection of these samples taken at different temperature is exhibited in Figure 2b. The samples become turbid close to the transition temperature, then, upon heating the formation of macro-aggregates was observed regardless of the macroCTA parent or architecture. At this point, hydrophobic interactions predominated in the system, enhanced by the strong hydrophobicity of the PCAE segment. Whittaker and co-workers [11] studied star PNIPAM polymer solutions and found that above the LCST, the polymer precipitated completely over a 12–24 h period. In the present work, the precipitation takes minutes because the PCAE block adds hydrophobic character to the system. Moreover, in many reports the presence of PEG enhances the stability of the copolymer system [29], nevertheless, here, the mentioned copolymers whether or not containing a hydrophilic segment follow the same behavior (Figure 2).

Stability of micellar aggregates is crucial for drug delivery application; accordingly, the copolymers synthesized in this part, could not be used as nanocarriers, due to the poor stability observed above their transition temperatures. Nevertheless, this behavior would be an advantage in other applications, for example, resolution of racemic mixtures using star-like polymers having optically active arms [35].

##### 3.4.2. pH/Thermosensitivity Behavior of Block Copolymers Containing Acrylic Acid

**Figure 3:**
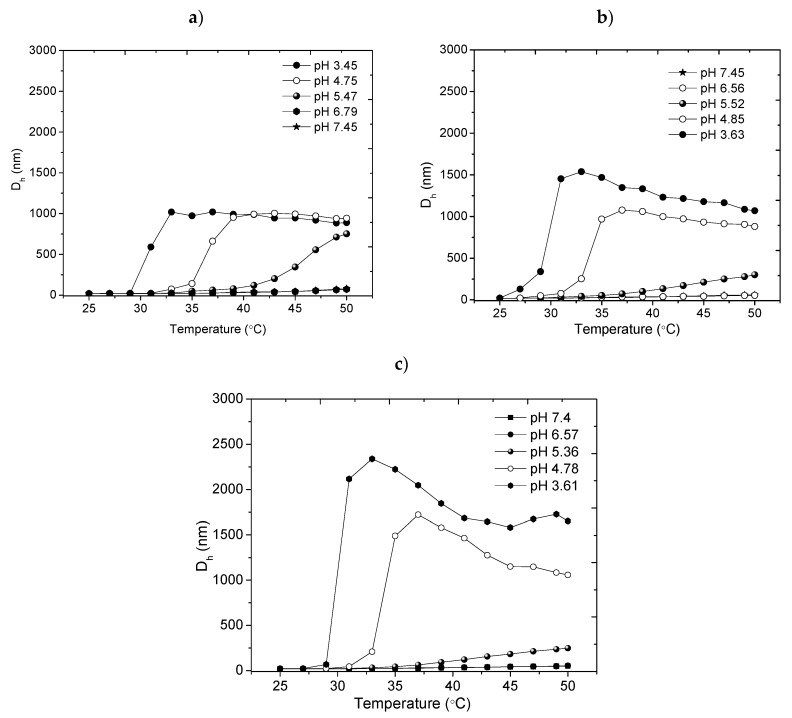
DLS measurements (*c* = 0.5 mg/mL in PBS). Evolution of the *D_h_* of (**a**) PAAc_2%_-*co*-PNIPAM_147_-*b*-PCAE_3_-*b*-PEG_45_-*b*-PCAE_3_-*b*-PNIPAM_147_-*co*-PAAc_2%_, (**b**) (GE_7_-*b*-PCAE_4_-*b*-PNIPAM_59_-*co*-PAAc_2%_)_3_ and (**c**) (PCAE_2_-*b*-PNIPAM_93_-*co*-PAAc_2%_)_4_ copolymers as a function of the temperature.

For pH-responsive copolymers containing acrylic acid (2% in feed), a decrease in the transition temperature was observed at lower values of pH. For instance, (PCAE_2_-*b*-PNIPAM_93_-*co*-PAAc_2%_)_3_ copolymer shows an LCST of 29 and 31 for pH values of 3.61 and 4.78 respectively. For the sample (GE_7_-*b*-PCAE_4_-*b*-PNIPAM_59_-*co*-PAAc_2%_)_3_ the LCST was 27 and 31 °C for pH values of 3.63 and 4.85 respectively. For the sample PAAc_2%_-*co*-PNIPAM_147_-*b*-PCAE_3_-*b*-PEG_45_-*b*-PCAE_3_-*b*-PNIPAM_147_-*co*-PAAc_2%_ the transition temperature was 27 and 35 °C for pH values of 3.45 and 4.75 respectively (Figure 3). The trend observed in these copolymers indicates a sharp phase transition for PNIPAM chains revealing a faster dehydration process. The LCST values in two samples were lower by 2 °C as compared with the copolymers without PAAc described above, which suggests that the PAAc content in the copolymer modifies the environment of the amide group of PNIPAM through hydrogen bonding with the protonated carboxylate groups. Above the LCST, a visual inspection of these samples reveals the formation of macro-aggregates (the same behavior as the copolymers without AAc described above). When the DLS measurements were carried out at higher values of pH (5.36 to 5.52) and above the transition temperature, a drastic decrease in the *D_h_* was observed. The trend observed in Figure 3 is consistent for all the copolymers; it can be assumed that the partial formation of carboxylate and its interaction with the carbonyl group in PNIPAM affected the dehydration process of the chains.

**Figure 4:**
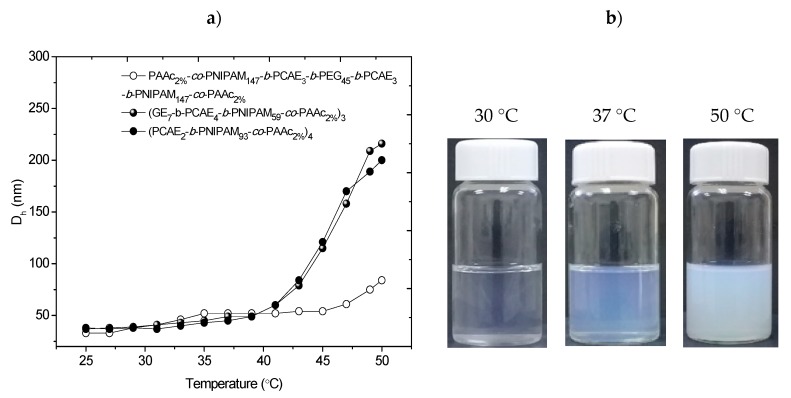
DLS measurements (*c* = 7 mg/mL in PBS). (**a**) Evolution of the *D_h_* of PAAc_2%_-*co*-PNIPAM_147_-*b*-PCAE_3_-*b*-PEG_45_-*b*-PCAE_3_-*b*-PNIPAM_147_-*co*-PAAc_2%_, (GE_7_-*b*-PCAE_4_-*b*-PNIPAM_59_-*co*-PAAc_2%_)_3_ and (PCAE_2_-*b*-PNIPAM_93_-*co*-PAAc_2%_)_4_ copolymers as a function of the temperature. (**b**) Representative pictures of (GE_7_-*b*-PCAE_4_-*b*-PNIPAM_59_-*co*-PAAc_2%_)_3_ copolymers taken at different temperatures.

Nevertheless, the *D_h_* remained almost unchanged at pH values ranged between ~6.50 to 7.45; the *D_h_* at 50 °C is ~60 nm (40 nm more than at 25 °C). The LCST is practically not observed. Consequently, the ionized carboxylate from PAAc produced strong hydrophilic character in the copolymer making difficult the dehydration process in the PNIPAM chains; this behavior is contrary to the copolymers without PAAc described above. The self-assembled behavior of the copolymers was also studied using 7.0 mg/mL in PBS. Analysis of DLS (Figure 4) revealed that the *D_h_* ranged from 25 to 35 nm below the LCST, suggesting the formation of compact aggregates; as can be observed—the size remains almost unchanged if compared at 0.5 mg/mL. Moreover, the evolution of *D_h_* with the temperature is displayed in Figure 4. For the samples (GE_7_-*b*-PCAE_4_-*b*-PNIPAM_59_-*co*-PAAc_2%_)_3_ and (PCAE_2_-*b*-PNIPAM_93_-*co*-PAAc_2%_)_4_ (Figure 4), an important increase was observed in the *D_h_* at 41 °C but the sharp transition observed for the copolymers without PAAc was absent here. Moreover, the *D_h_* gradually increases upon heating; at 50 °C the samples acquire a “milky” appearance. Interestingly, the samples exhibited very good stability at 37 °C and no evidence of precipitation over a 12 h period. After cooling, the dispersions become transparent again. For instance, photographs of the (GE_7_-*b*-PCAE_4_-*b*-PNIPAM_59_-*co*-PAAc_2%_)_3_ copolymer are exhibited in Figure 4b. The sample PAAc_2%_-*co*-PNIPAM_147_-*b*-PCAE_3_-*b*-PEG_45_-*b*-PCAE_3_-*b*-PNIPAM_147_-*co*-PAAc_2%_ shows only a slightly increase in *D_h_* above the transition temperature, probably due to the low content of PCAE (1.7%) in the copolymer (Figure 4) and turbidity at 50 °C is barely observable. In addition to this, there are many reports with thermosensitive amphiphilic copolymers forming micellar aggregates below the LCST; but in the present work, the copolymers self-assembled in compact aggregates with a tight core of PCAE.

**Scheme 5:**
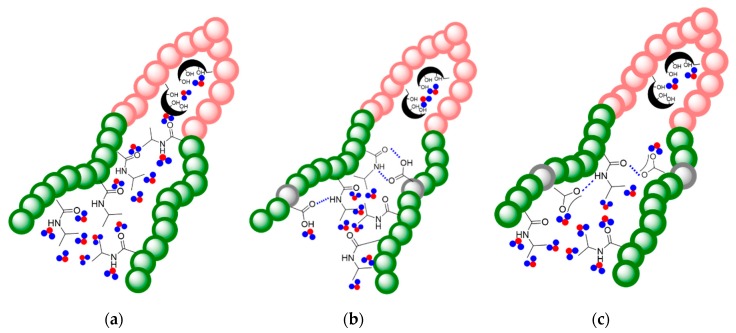
Hydrated block copolymers below the LCST (**a**) copolymers without PAAc acid (**b**) copolymers containing PAAc at lower value of pH and (**c**) copolymers containing PAAc at a pH value of 7.45.

The hydrated block copolymers and their molecular/ionic interactions below the LCST are represented in Scheme 5: (a) copolymers without PAAc acid, (b) copolymers containing PAAc at low pH value, and (c) copolymers containing PAAc at pH value of 7.45. Note that only the PNIPAM and PCAE blocks are represented to gain clarity.

## 4. Conclusions

In summary, well-defined PCAE-*b*-PNIPAM and PCAE-*b*-PNIPAM-*co*-PAAc copolymers having different topologies were synthesized by the RAFT (R-RAFT approach). The structure of both macroCTAs and copolymers were characterized by ^1^H NMR spectroscopy. The macroCTAs and copolymers showed monomodal distribution and acceptable dispersity values based on GPC measurements. The content of PCAE in the copolymers (1.7% to 5.7%) decreases the LCST of PNIPAM by approximately 3 °C. The diameters of the star and linear copolymers ranging from 18 to 30 nm obtained by DLS suggest that all copolymers without distinction, are self-assembled in compact aggregates with a PCAE core. The PCAE-*b*-PNIPAM copolymers were unstable in PBS solution above the LCST. The PCAE-*b*-PNIPAM-*co*-PAAc copolymers showed a faster precipitation above the LCST at lower values of pH. On the contrary, copolymer solutions containing PAAc showed great stability at 37 °C and higher pH values for a longer time period. The self-assembly of the linear and star copolymers showed similar behavior although the topology and the block sequence of the polymers in the chain were significantly different.

## Figures and Tables

**Table 1:** Results of RAFT polymerization of CAE in DMF at 68 °C in the presence of CTAs 1 to 3 trithiocarbonate-type *.

CTA	MacroCTA	Molar Ratio [CAE]/[CTA]/[AIBN]	Time (min)	Conv. ^a^ (%)	*Mn*_theo_^b^ (g/mol)	*Mn*_GPC_^c^ (g/mol)	*Ɖ* ^c^	*Mn*_NMR_^d^ (g/mol)
**1**	PCAE_3_-*b*-PEG_45_-*b*-PCAE_3_	74/5/1	300	65	7572	6036	1.10	5502
**1**	PCAE_5_-*b*-PEG_45_-*b*-PCAE_5_	69/5/1	360	55	6543	8487	1.21	7769
**2**	(GE_7_-*b*-PCAE_4_)_3_	71/5/1	120	60	6242	8519	1.06	7333
**3**	(PCAE_2_)_4_	80/5/1	90	44	5032	7200	1.12	5079

^a^ Determined gravimetrically. ^b^ Calculated using equation: $M_{n,\mathrm{CAL}}=\left(\frac{\left[M\right]}{\left[\mathrm{CTA}\right]}\cdot M_{\mathrm{PCAE}}\cdot \mathrm{Conv}.\right)+M_{\mathrm{CTA}}$. ^c^ Determined by GPC in THF at 35 °C with RI and LS detectors using polystyrene linear standards for calibration of LS detector (d*n*/d*c* = 0.093 mL/g). ^d^ Determined by ^1^H NMR. * All samples were acetylated for GPC analyses. * The subscript numbers represented the repeating units of each block determined by ^1^H NMR.

**Table 2:** Results for RAFT polymerization of PNIPAAm or PNIPAM-*co*-PAAc in the presence of the macroCTAs 1 to 3 in DMF at 68 °C *.

MacroCTA	Copolymer	Molar Ratio [NIPAM]/[AAc]/[macroCTA]/[AIBN]	Conv. ^a^ (%)	*Mn*_NMR_^b^ (g/mol)	*Mn*_GPC_^c^ (g/mol)	*Ɖ* ^c^
1	PNIPAM_120_-*b*-PCAE_3_-*b*-PEG_45_-*b*-PCAE_3_-*b*-PNIPAM_120_	333/0/2.5/1	76	33,109	25,620	1.3
1	PNIPAM_87_-*b*-PCAE_5_-*b*-PEG_45_-*b*-PCAE_5_-*b*-PNIPAM_87_	323/0/2.5/1	45	39,299	27,400	1.4
1	PAAc_2%_-*co*-PNIPAM_147_-*b*-PCAE_3_-*b*-PEG_45_-*b*-PCAE_3_-*b*-PNIPAM_147_-*co*-PAAc_2%_	326/7/2.5/1	72	26,664	21,440	1.3
2	(GE_7_-*b*-PCAE_4_-*b*-PNIPAM_79_)_3_	980/0/2.5/0.25	75	34,500	32,660	1.4
2	(GE_7_-*b*-PCAE_4_-*b*-PNIPAM_59_-*co*-PAAc_2%_)_3_	960/20/2.5/0.25	60	27,926	24,200	1.3
3	(PCAE_2_-*b*-PNIPAM_75_)_4_	500/0/1/0.25	81	-	41,400	1.37
3	(PCAE_2_-*b*-PNIPAM_93_-*co*-PAAc_2%_)_4_	490/10/1/0.25	73	-	49,400	1.28

^a^ Determined gravimetrically. ^b^ Determined by ^1^H NMR. ^c^ Determined by GPC in THF at 35 °C with RI and LS detectors using polystyrene linear standards for calibration of LS detector (d*n*/d*c* = 0.093 mL/g). * The subscript numbers represented the repeating units of each block.

## Supplementary Materials

The following are available online at https://www.mdpi.com/2073-4360/11/11/1859/s1.
