# Supplementary material for: Thermo/pH Responsive Star and Linear Copolymers Containing a Cholic Acid-Derived Monomer, N-Isopropylacrylamide and Acrylic Acid: Synthesis and Solution Properties
Source: Polymers (Basel). 2019 Nov 11;11(11):1859. doi: 10.3390/polym11111859 (PMC6918292; doi:10.3390/polym11111859)
Supplement: Supplementary file 1 [file polymers-11-01859-s001.pdf]

Supporting Information

Thermo/pH responsive star and linear copolymers containing a cholic acid-derived monomer, *N*-Isopropylacrylamide and acrylic acid: synthesis and study of solution behavior

Ana Castro-Hernández and Norma Aidé Cortez-Lemus*

Centro de Graduados e Investigación en Química. Tecnológico Nacional de México/Instituto Tecnológico de Tijuana. A. P. 1166. Tijuana, B. C., C.P. 22000, México.

E-mail: ncortez@tectijuana.mx

Index

Contents	………………………………………………………………………..	Page
Synthesis of a cholic acid-derived monomer CAE		2
**Figure S1**.	^{1}H-NMR (400 MHz) spectrum of 2-(acryloyloxy)ethyl cholate (CAE) in $CDCl_3$.	3
Synthesis of CTA's **1** to **3**		3
Synthesis of CTA-**2**		3
**Figure S2**	^{1}H-NMR (400 MHz) spectrum of bromide precursor from CTA-**1**-Br in $CDCl_3$.	5
**Figure S3**	^{13}C-NMR (100 MHz) spectrum of bromide precursor from CTA-**1-Br** in $CDCl_3$.	6
**Figure S4**	^{1}H-NMR (400 MHz) spectrum of CTA-**1** in $CDCl_3$.	6
**Figure S5**	^{13}C-NMR (100 MHz) spectrum of CTA-**1** in $CDCl_3$.	7
**Figure S6**	^{1}H-NMR (400 MHz) spectrum of bromide precursor from CTA-**2**-Br in $CDCl_3$.	8
**Figure S7**	^{13}C-NMR (100 MHz) spectrum of bromide precursor from CTA-**2**-Br in $CDCl_3$.	9
**Figure S8**	^{1}H-NMR (400 MHz) spectrum of CTA-**2** in $CDCl_3$.	9
**Figure S9**	^{13}C-NMR (100 MHz) spectrum of CTA-**2** in $CDCl_3$.	10
**Figure S10**	^{1}H-NMR (400 MHz) spectrum of tetrafunctional bromide precursor CTA-**3-**Br in $CDCl_3$.	11
**Figure S11**	^{13}C-NMR (100 MHz) spectrum of tetrafunctional bromide precursor CTA-**3**-Br in $CDCl_3$	11
**Figure S12**	^{1}H-NMR (400 MHz) spectrum of CTA-**3** in $CDCl_3$.	12
**Figure S13**	^{13}C-NMR (100 MHz) spectrum of CTA-**3** in $CDCl_3$.	13
Synthesis of macroCTA's (polymerization of PCAE using the CTA's **1** to **3** by RAFT)		14
Synthesis of $PCAE_3$-*b*-PEG_{45}-*b*-$PCAE_3$ or macroCTA-**1**		14
**Figure S14**	^{1}H-NMR (400 MHz) spectrum of macroCTA-**1** in $CDCl_3$.	15
Synthesis of (GE_7-*b*-$PCAE_4$)$_3$ or macroCTA-**2**		15

Figure S15 ^{1}H-NMR (400 MHz) spectrum of macroCTA-**2** in $CDCl_3$. 16
Synthesis of $(PCAE_2)_4$ or macroCTA-**3** 16
Figure S16 ^{1}H-NMR (400 MHz) spectrum of macroCTA-**3** in $CDCl_3/CD_3OD$. 17
Chain extension polymerization of NIPAM with or without acrylic acid in presence of the macroCTA's 18
Figure S17 ^{1}H-NMR (400 MHz) spectrum of $PNIPAM_{120}$-*b*-$PCAE_3$-*b*-PEG_{45}-*b*-$PCAE_3$-*b*-$PNIPAM_{120}$ in $CDCl_3$. 18
Figure S18 ^{1}H-NMR (400 MHz) spectrum of $PAAc_{2\%}$-*co*-$PNIPAM_{147}$-*b*-$PCAE_3$-*b*-PEG_{45}-*b*-$PCAE_3$-*b*-$PNIPAM_{147}$-*co*-$PAAc_{2\%}$ in $CDCl_3$. 19
Figure S19 ^{1}H-NMR (400 MHz) spectrum of $(GE_7$-*b*-$PCAE_4$-*b*-$PNIPAM_{79})_3$ in $CDCl_3$. 20
Figure S20 ^{1}H-NMR (400 MHz) spectrum of $(GE_7$-*b*-$PCAE_4$-*b*-$PNIPAM_{59}$-*co*-$AAc_{2\%})_3$ in $CDCl_3$. 20
Figure S21 ^{1}H-NMR (400 MHz) spectrum of $(PCAE_2$-*b*-$PNIPAM_{75})_4$ copolymer in $CDCl_3$. 21
Figure S22 ^{1}H-NMR (400 MHz) spectrum of $(PCAE_2$-*b*-$PNIPAM_{93}$-*co*-$AAc_{2\%})_4$ copolymer in $CDCl_3$. 21
Figure S23 Hydrodynamic diameter (D_h) for $(GE_7$-*b*-$PCAE_4$-*b*-$PNIPAM_{79})_3$ copolymer at different concentrations (0.5 to 8 mg/mL in PBS) by DLS at 25 ºC. 22
References 22

EXPERIMENTAL SECTION

Synthesis of a cholic acid-derived monomer CAE. The synthesis of CAE was carried out following the methodology reported by De and co-workers [1].

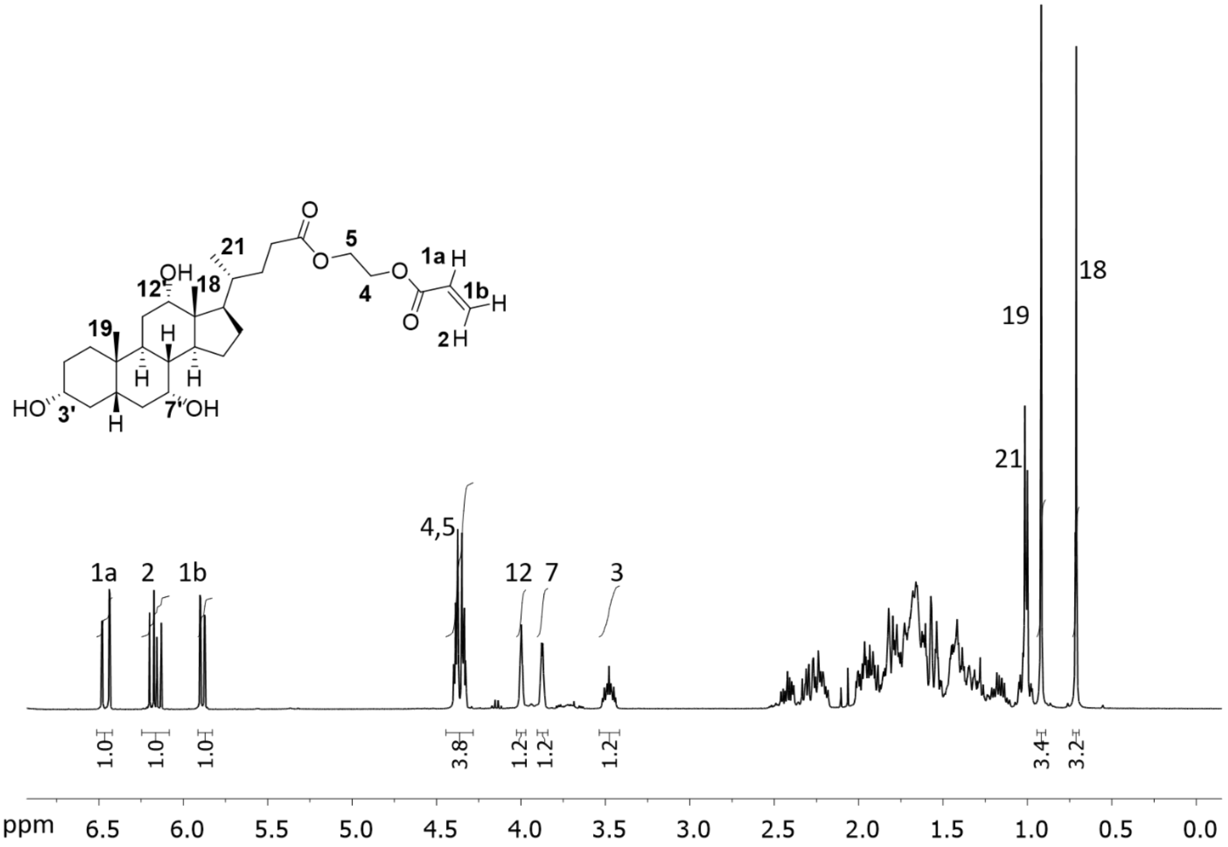

Figure S1. ^{1}H-NMR (400 MHz) spectrum of 2-(acryloyloxy)ethyl cholate (CAE) in $CDCl_3$.

2-(acryloyloxy)ethyl cholate (CAE). ^{1}H NMR ($CDCl_3$, 400 MHz), δ (ppm): 6.44-6.48 (C***H***$_2$=CHCO trans, 1H, dd, J_1=17.2 Hz, J_2=1.2), 6.20-6.13 (COC***H***=CH_2, 1H, dd, J_1=17.2 Hz, J_2=10.40), 5.90-5.87 (C***H***$_2$=CHCO cis, 1H, dd, J_1=10.4 Hz, J_2=1.2), 4.40-4.33 (COO-C***H***$_2$C***H***$_2$-OCO, 4H, m), 3.99 (12'-C***H***, 1H, m), 3.88 (7'-C***H***, 1H, m), 3.48 (3'-C***H***, 1H, m), 1.01 (21'-C***H***$_3$, 3H, d, *J*=6.28), 0.92 (19'-C***H***$_3$, 3H, s) and 0.71 (18'-C***H***$_3$, 3H, s), R_f = 0.30 (1:1 ethyl acetate:CH_2Cl_2).

Synthesis of CTA's **1** to **3**

Synthesis of CTA-**2**. Step 1: *Synthesis of bromide precursor*. Glycerol ethoxylate (4 g, 0.004 mol) was dissolved in dry THF (45 mL) and pyridine was then added (1.105 g, 0.014 mol). This solution was ice-cooled and 2-bromopropionyl bromide (1.46 mL, 0.014 mol in

5 mL of dry THF) was added dropwise. After complete addition, the mixture reaction was maintained in ice for 1 h. The solution was stirred at room temperature overnight. The amine salt formed was filtered off and the filtrated was concentrated under reduced pressure. Next, the product was dissolved in 50 mL of CH_2Cl_2 and HCl (30 mL, 0.01 M) was added to the solution (X3). Next, $NaHCO_3$ (30 mL, 0.01 M). The reaction mixture was dried with Na_2SO_4. After evaporation of solvent under reduced pressure, the bromide precursor of CTA-**2** was purified using column chromatography on silica gel (CH_2Cl_2:CH_3OH, 4:1). 2.5 g of yellowish liquid was obtained.

Step 2: Dodecanethiol (0.86 g, 4.26 mmol) was dissolved in THF (15 mL) and triethylamine (0.32 g, 4.26 mmol) was added to the reaction mixture. After 10 min, CS_2 was added dropwise (0.61 mL, 4.26 mmol). Next, the bromide trifunctional precursor was added to the yellow solution (1.5 g, 1.065 mmol) and stirred overnight at room temperature. After remove the amine salt, the yellow filtrated was evaporate under reduced pressure. Finally, the trithiocarbonate was purified using column chromatography on silica gel (CH_2Cl_2:CH_3OH, 5:1). 2.5 g of yellow oil thick was obtained.

Note: The synthesis of CTA **1** and **3** was following this methodology but using poly(ethylene glycol) (2000 g/mol) and PERT respectively, for the synthesis of the brominate precursors.

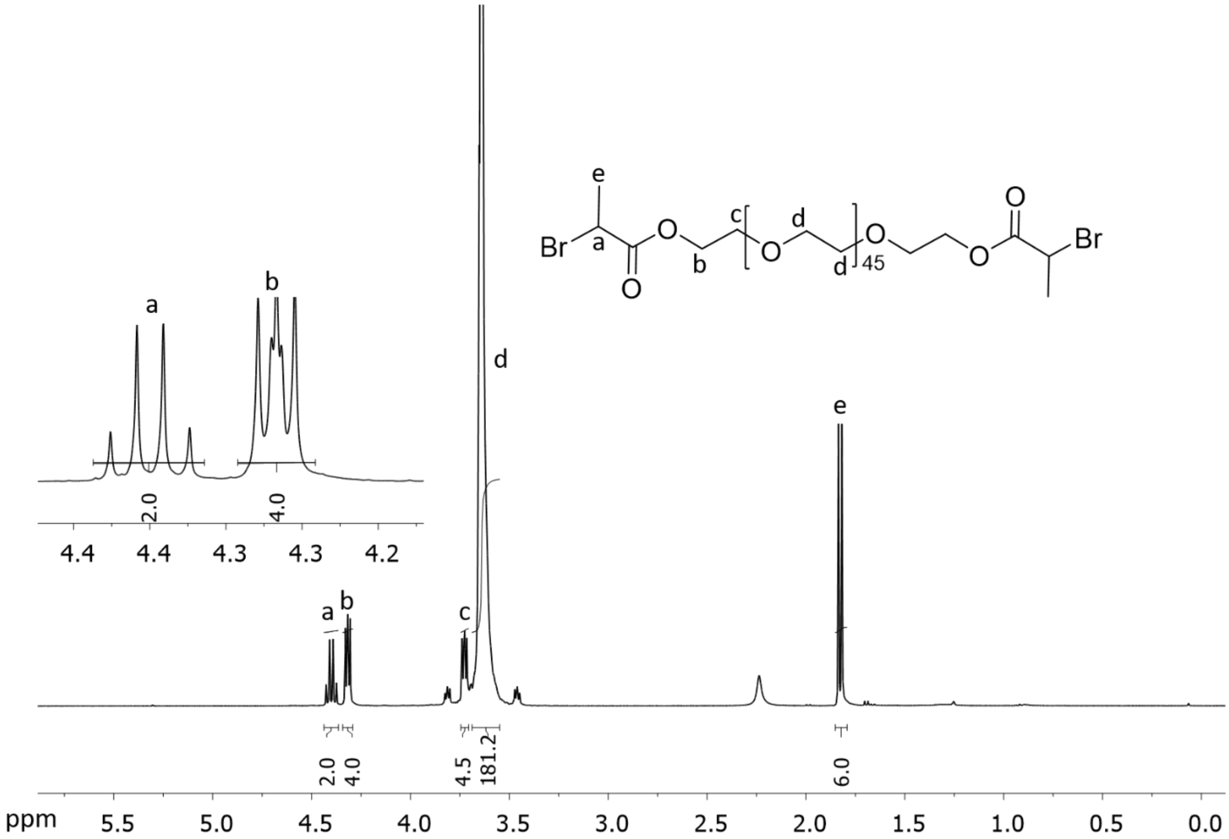

Figure S2. ^{1}H-NMR (400 MHz) spectrum of bromide precursor from CTA-**1**-Br in $CDCl_3$.

CTA-**1**-Br. ^{1}H NMR ($CDCl_3$, 400 MHz), δ (ppm): 4.43-4.36 ($CH_3C\boldsymbol{H}Br$, 2H, q, *J*= 7.2 Hz and *J*= 6.8 Hz), 4.32 (O=CO-C$\boldsymbol{H}_2$-CH_2-, 4H, t, *J*= 4.8 Hz), 3.73 (-CH_2-C$\boldsymbol{H}_2$-O, 4H, t), 3.66 (-C$\boldsymbol{H}_2$C$\boldsymbol{H}_2$-O, 181H, s), 1.82 (C$\boldsymbol{H}_3$CHBr, 6H, d, *J*= 7.2 Hz) (See Fig. S2). ^{13}C NMR ($CDCl_3$): 170.2 (***C***=O), 70.6 (-***C***H_2***C***H_2-O), 68.8 (O=CO-***C***H_2-CH_2-, 65.0 (-CH_2-***C***H_2-O), 39.9 (CH_3***C***HBr), 21.6 (***C***H_3CHBr) (See Fig. S3).

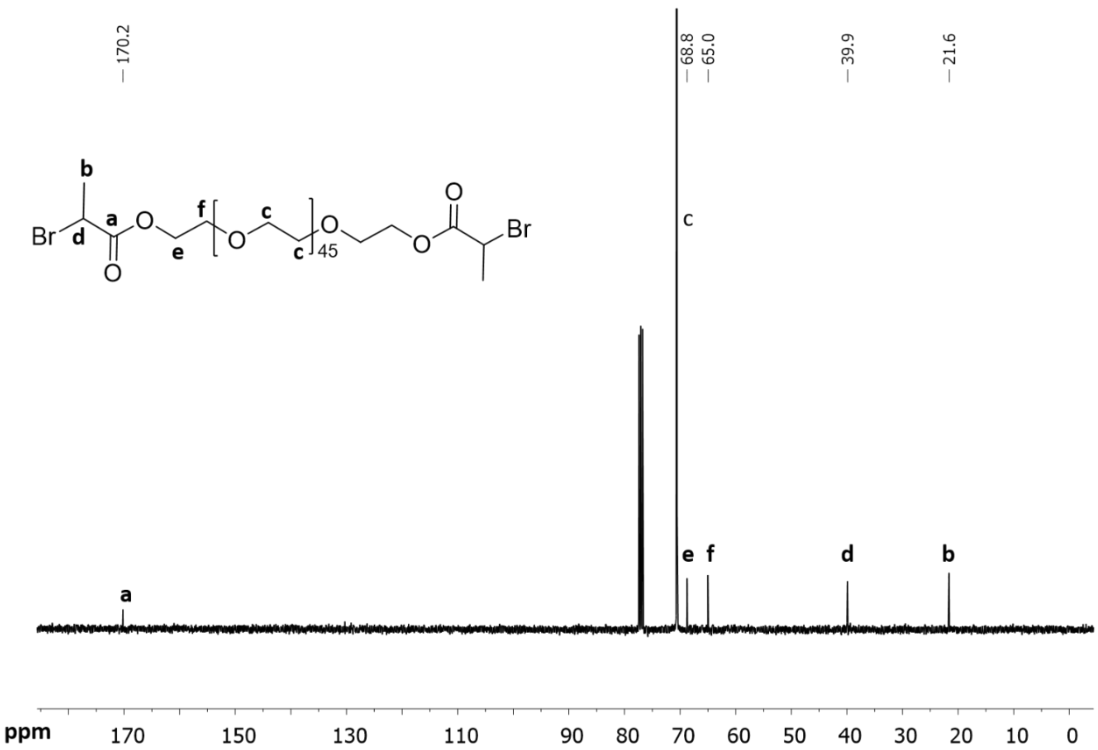

Figure S3. ^{13}C-NMR (100 MHz) spectrum of bromide precursor from CTA-**1-**Br in $CDCl_3$.

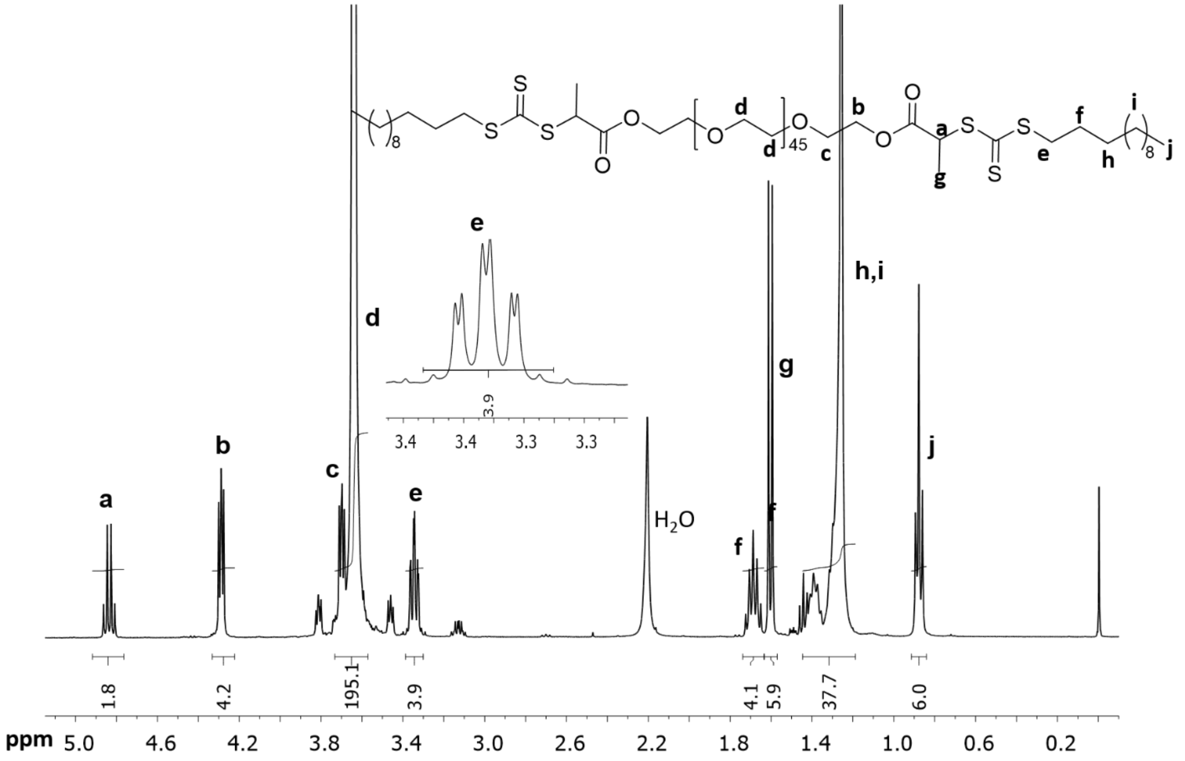

Figure S4. ^{1}H-NMR (400 MHz) spectrum of CTA-**1** in $CDCl_3$.

CTA-**1.** ^{1}H NMR ($CDCl_3$, 400 MHz), δ (ppm): 4.88-4.79 (CH_3-C***H***-S, q, 2H, *J*= 7.2 Hz and *J*= 7.2 Hz), 4.29 (-(CH_2-CH_2)-O-CH_2-C***H***$_2$-O-, 4H, t, *J*= 4.8 Hz), 3.70 (CH_2-CH_2)-O-C***H***$_2$-CH_2-O-, t, 4H), 3.66 (-C***H***$_2$-C***H***$_2$-O, s, 195 H), 3.38-3.31 (-CH_2C***H***$_2$-S, td, 4H, *J*= 2.0 Hz and *J*= 5.2 Hz), 1.69 (-S-CH_2-C***H***$_2$, q, 4H, *J*= 7.2 Hz), 1.60 (C***H***$_3$-CH-S, d, 6H, *J*= 7.2 Hz), 1.47-1.19 (-C***H***$_2$C***H***$_2$- 38H), 0.88 (C***H***$_3$$CH_2$-, 6H, t, *J*= 6.4 Hz) (See Fig. S4). [13]C NMR ($CDCl_3$): 221.9 (***C***=S), 171.0 (***C***=O), 70.6 (-***C***H_2***C***H_2-O), 68.8 (O=CO-***C***H_2-CH_2-), 64.9 (O=CO-CH_2-***C***H_2-O-) 16.9 (***C***H_3CH-S)**,** 14.0 (***C***$H_3$$CH_2$-) (See Fig. S5).

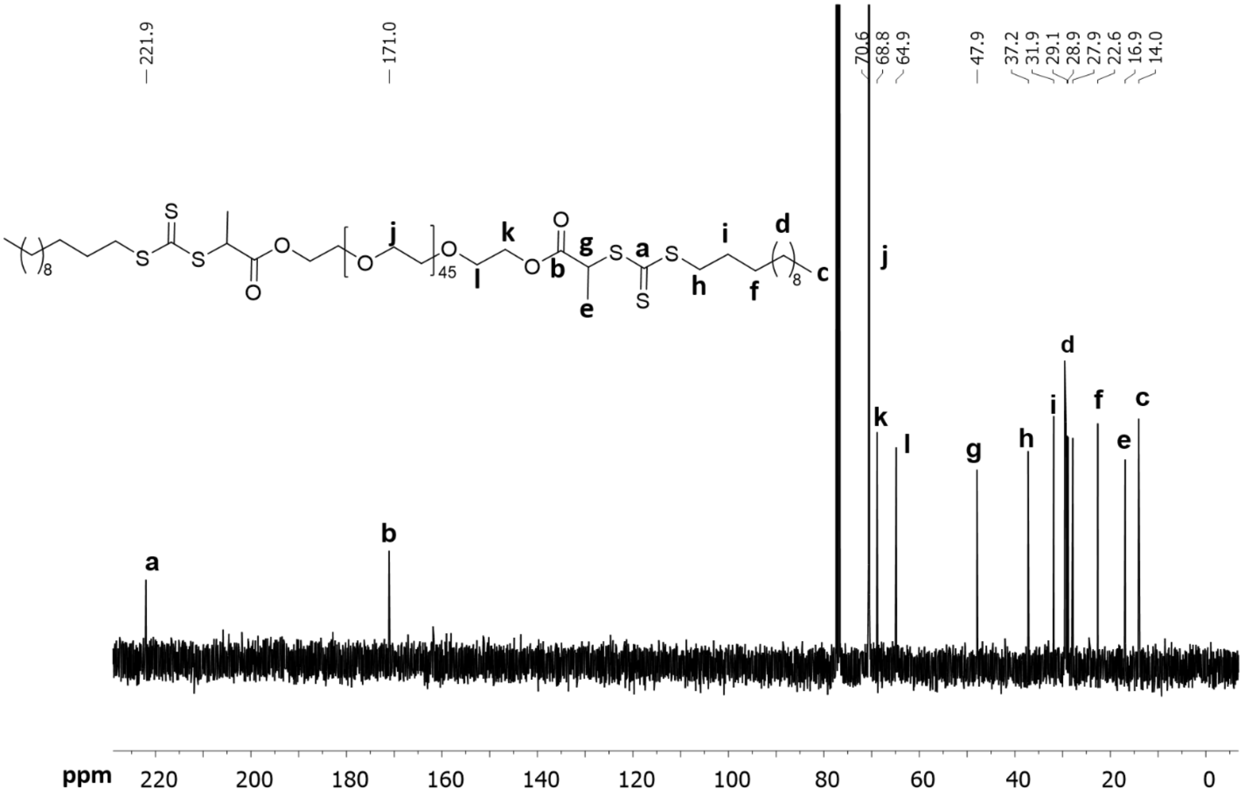

Figure S5. ^{13}C-NMR (100 MHz) spectrum of CTA-**1** in $CDCl_3$.

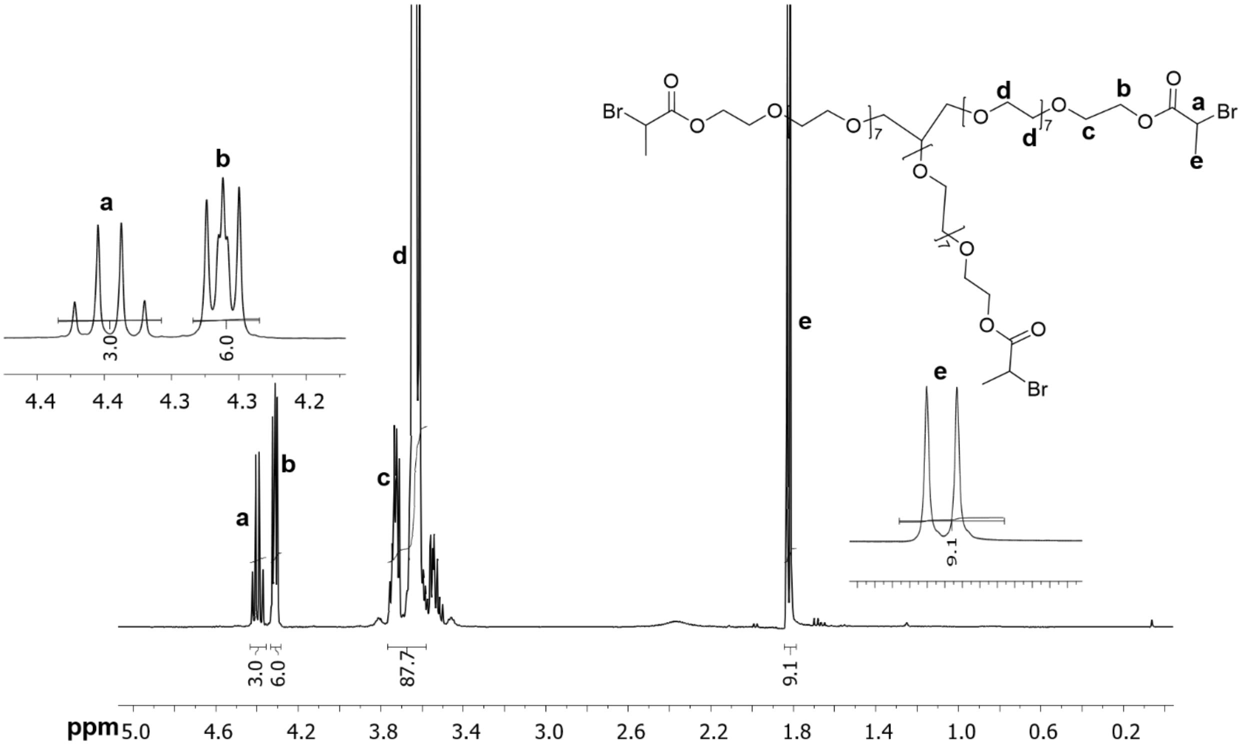

Figure S6. ^{1}H-NMR (400 MHz) spectrum of bromide precursor from CTA-**2**-Br in CDCl$_3$.

CTA-**2**-Br. ^{1}H NMR (CDCl$_3$, 400 MHz), δ (ppm): 4.43-4.36 (CH$_3$C***H***Br, 3H, q, *J*= 6.8 Hz and *J*= 7.2 Hz), 4.31 (-CH$_2$-CH$_2$-O-C***H***$_2$-, 6H, t, *J*= 4.8 Hz), 3.72 (-CH$_2$-C***H***$_2$-O-CO, 6H, m), 3.66 (-C***H***$_2$C***H***$_2$-O, 88H, s), 1.82 (C***H***$_3$CHBr, 9H, d, *J*= 6.8 Hz) (See Fig. S6). ^{13}C NMR (CDCl$_3$): 170.2 (***C***=O), 78.35 (***C***H), 69.70 (-***C***H$_2$***C***H$_2$-O), 68.70 (-O-***C***H$_2$-CH$_2$-), 65.0 (-O-CH$_2$-***C***H$_2$-), 39.9 (CH$_3$***C***HBr), 21.6 (***C***H$_3$CHBr) (See Fig. S7).

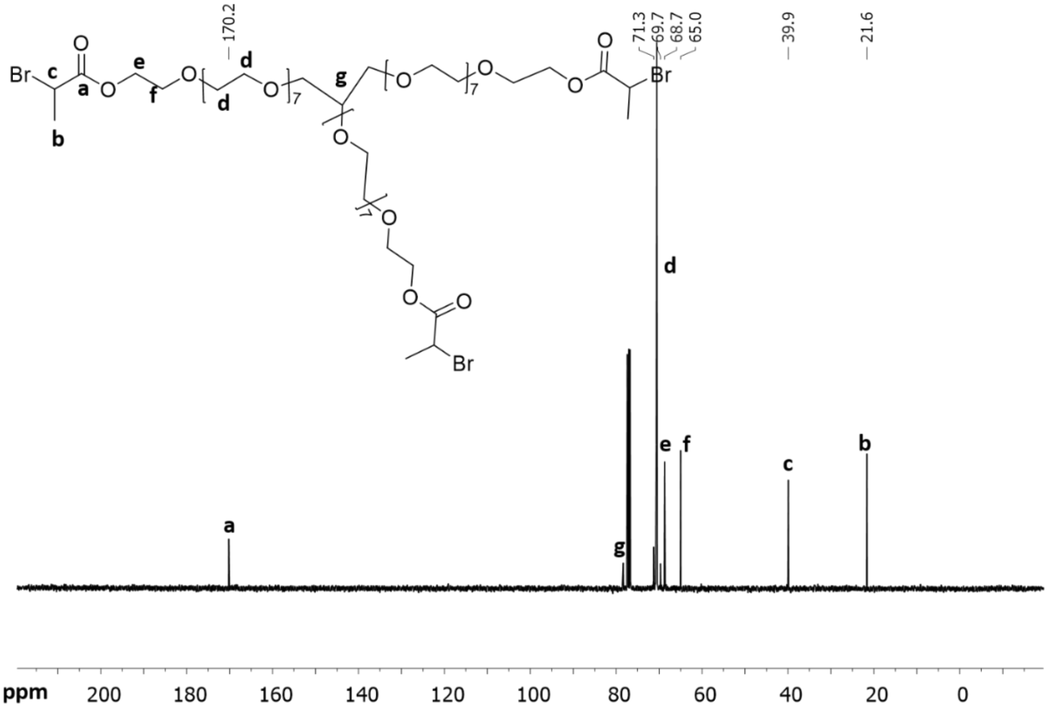

Figure S7. ^{13}C-NMR (100 MHz) spectrum of bromide precursor from CTA-**2**-Br in $CDCl_3$.

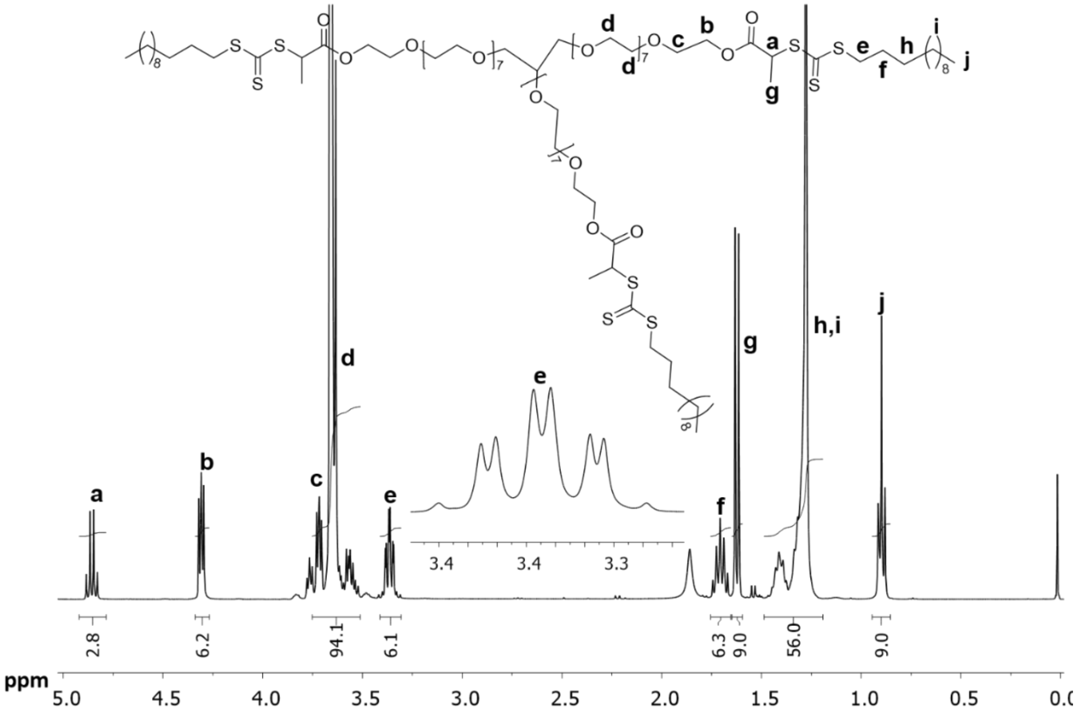

Figure S8. ^{1}H-NMR (400 MHz) spectrum of CTA-**2** in $CDCl_3$.

CTA-**2.** ^{1}H NMR ($CDCl_3$, 400 MHz), δ (ppm): 4.90-4.80 (CH_3-C***H***-S, 3H, q, *J*= 7.2 Hz and *J*= 7.2 Hz), 4.31 (-(CH_2-CH_2)-O-CH_2-C***H***$_2$-O-, 6H, t, *J*= 4.8 Hz), 3.72 (-(CH_2-CH_2)-O-C***H***$_2$-CH_2-O, 4H, t), 3.66 (-C***H***$_2$-C***H***$_2$-O, 94H, s), 3.36 (-CH_2-C***H***$_2$-S-, 6H, td, *J*= 4.8 Hz), 1.71 (-C***H***$_2$$CH_2$-S, q, 6H, *J*= 7.2 Hz), 1.62 (C***H***$_3$-CH-S, 9H, d, *J*= 7.2 Hz), 1.47-1.20 (-C***H***$_2$C***H***$_2$-**5**6H), 0.90 (C***H***$_3$$CH_2$-, 9H, t, *J*= 6.8 Hz) (See Fig. S8). [13]C NMR ($CDCl_3$): 221.9 (***C***=S), 171.1 (***C***=O), 70.6 (-***C***H_2***C***H_2-O),16.9 (***C***H_3CH-S)**,**14.0 (***C***$H_3$$CH_2$-) (See Fig. S9).

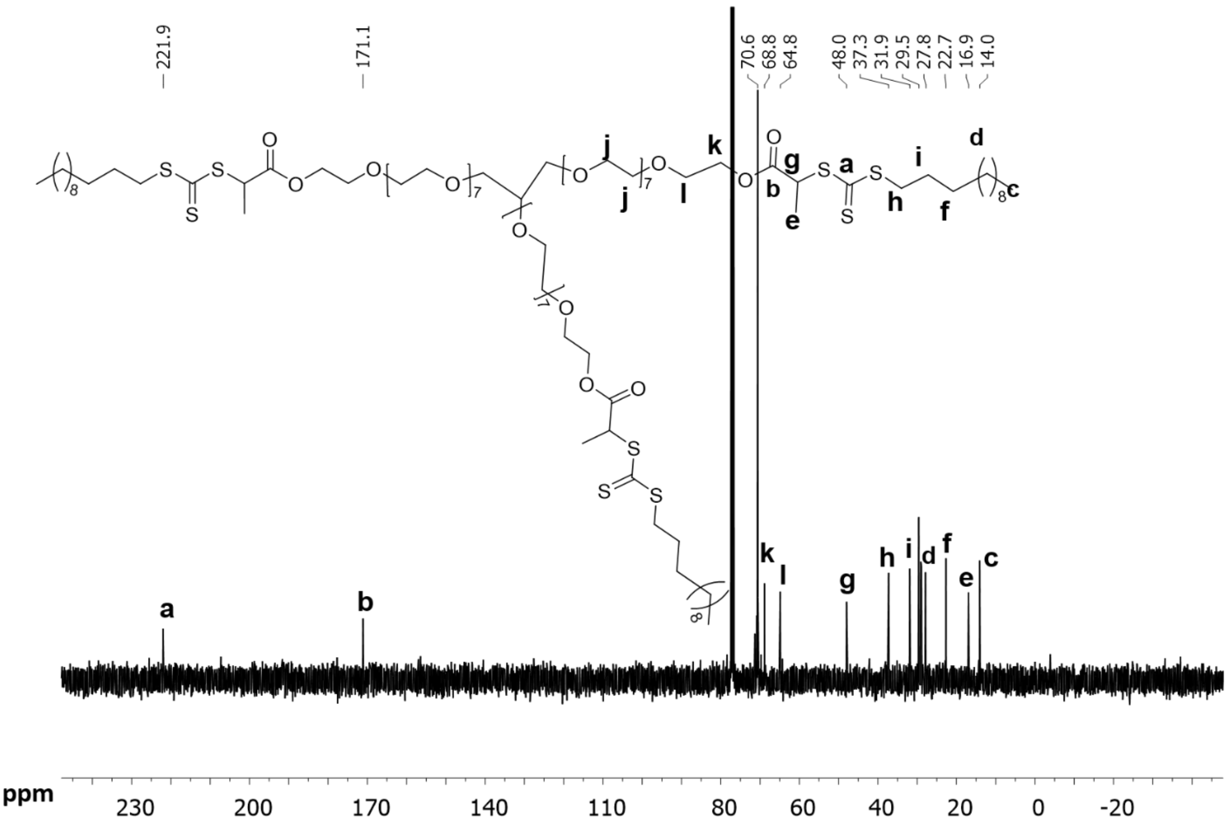

Figure S9. ^{13}C-NMR (100 MHz) spectrum of CTA-**2** in $CDCl_3$.

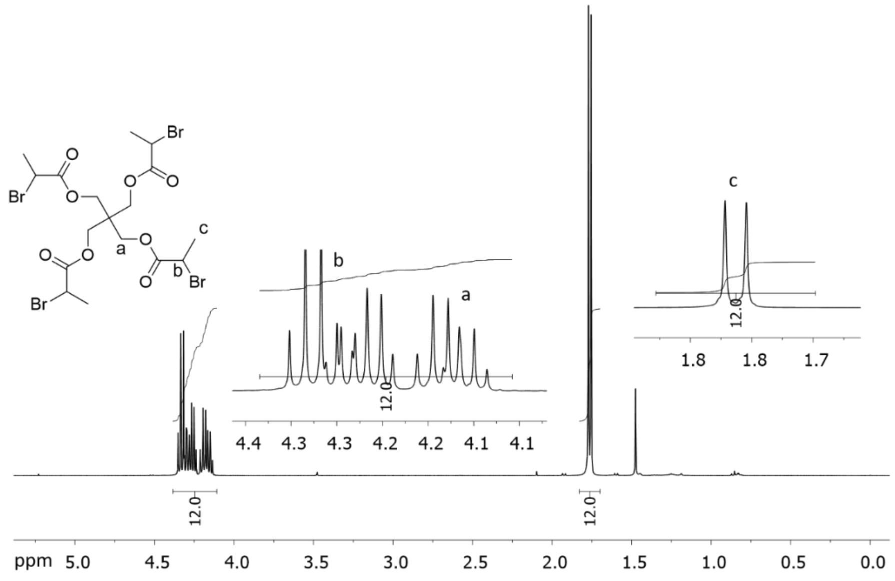

Figure S10. ^{1}H-NMR (400 MHz) spectrum of tetrafunctional bromide precursor CTA-**3-**Br in $CDCl_3$.

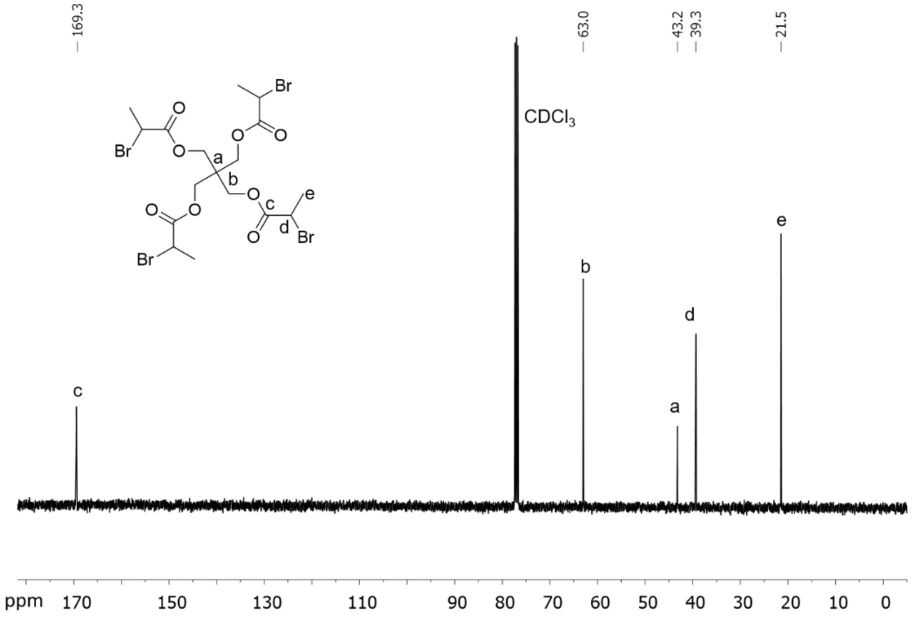

Figure S11. ^{13}C-NMR (100 MHz) spectrum of tetrafunctional bromide precursor CTA-**3**Br in $CDCl_3$.

CTA-**3**Br. Tetrafunctional bromide precursor. ^{1}H NMR ($CDCl_3$, 400 MHz), δ (ppm): 4.36-4.12 (C***H***$_2$-OCO and CH_3C***H***Br, 12H, m), 1.76 (C***H***$_3$CHBr, 12H, d, *J*=6.93) (See Fig. S10). ^{13}C NMR ($CDCl_3$): 169.3 (***C***=O), 63 (***C***H_2-O), 43.2 (-***C***-CH_2-O), 39.3 (CH_3***C***HBr), 21.5 (***C***H_3CHBr) (See Fig. S11). R_f = 0.24 (hexanes:CH_2Cl_2 1:1).

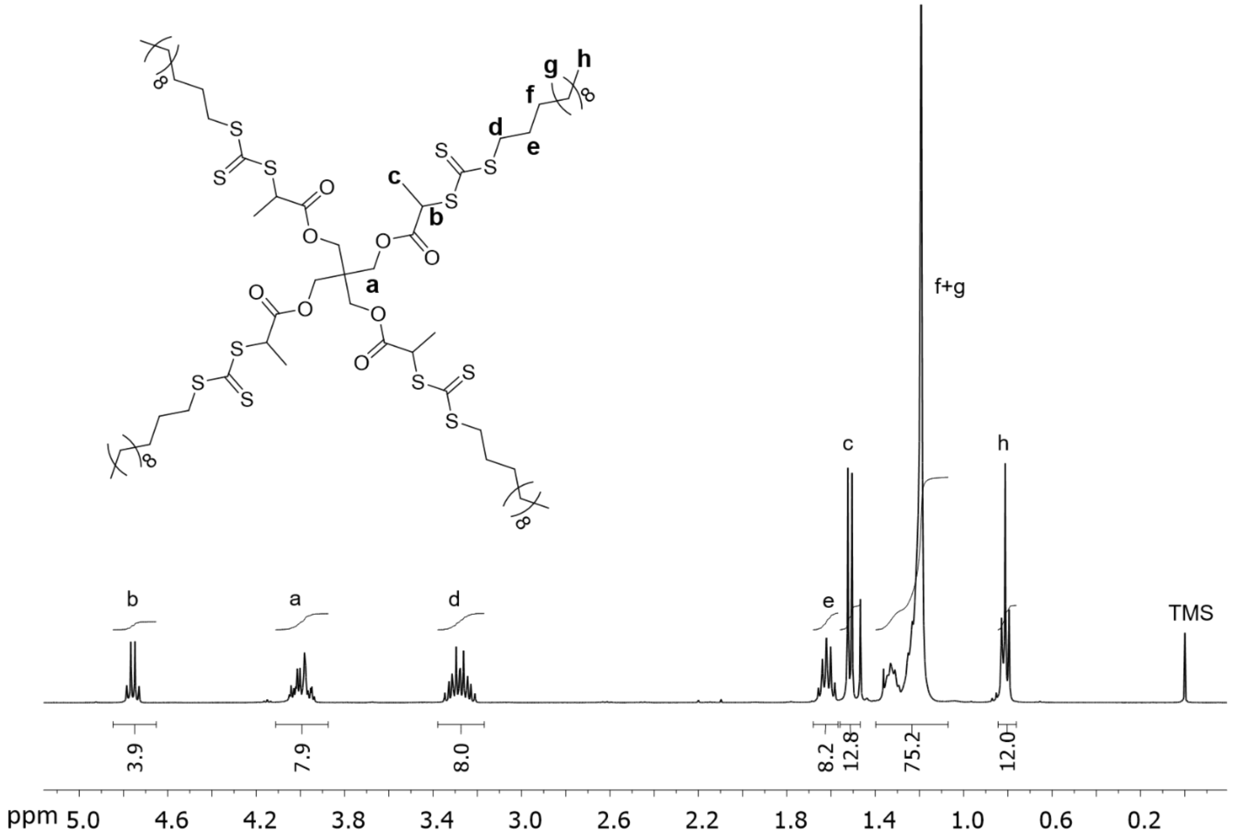

Figure S12. ^{1}H-NMR (400 MHz) spectrum of CTA-**3** in $CDCl_3$.

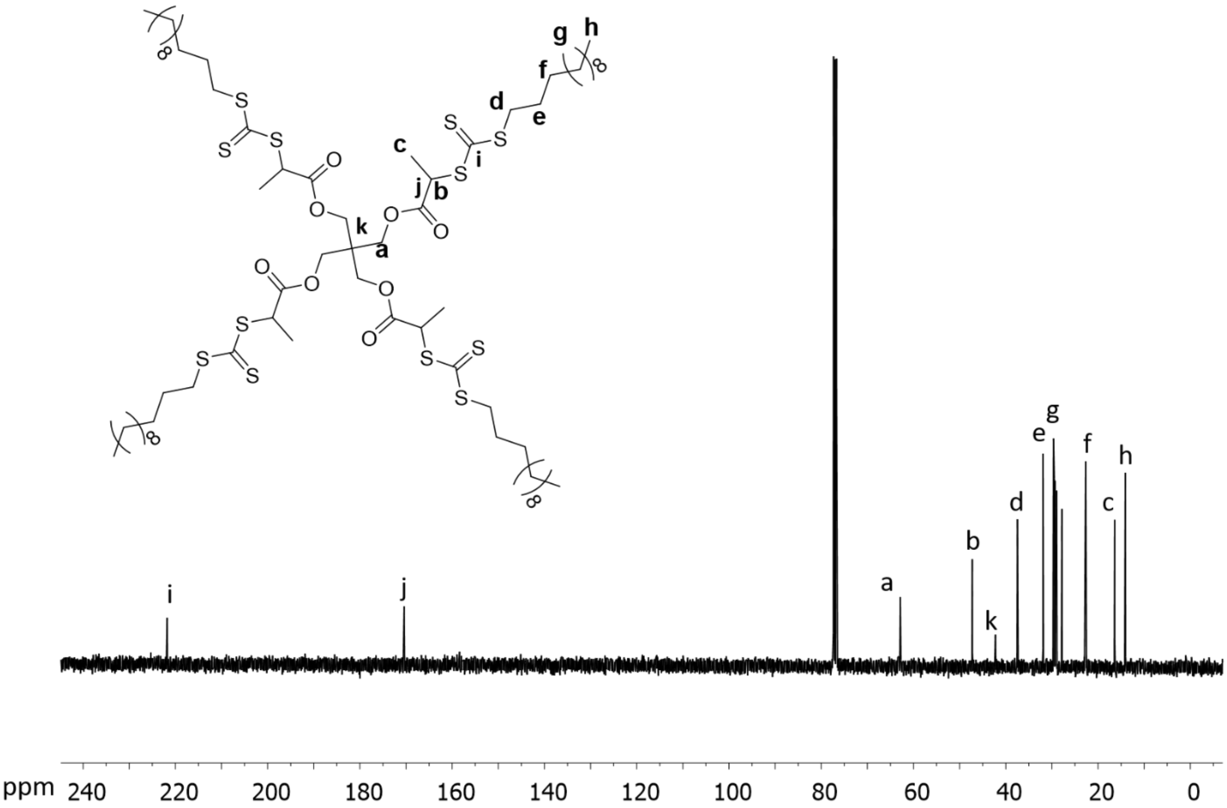

Figure S13. ^{13}C-NMR (100 MHz) spectrum of CTA-**3** in $CDCl_3$.

CTA-**3**, Tetrafunctional trithiocarbonate. ^{1}H NMR ($CDCl_3$, 400 MHz), δ (ppm): 4.77 (SCS-C***H***-CO, 4H, q, *J*=7.41 Hz), 3.99 (C***H***$_2$-OCO, 8H, m), 3.28 (C***H***$_2$-SCS, 8H, t, *J*=7.43 Hz), 1.62 (C***H***$_2$-CH$_2$-SCS, 8H, quintet, *J*=7.43 Hz), 1.51 (C***H***$_3$-CH-SCS, 12H, d, *J*=7.41 Hz), 1.36-1.19 (-(C***H***$_2$)$_8$-, 75H, m, aliphatic chain), 0.82 (-CH$_2$-C***H***$_3$, 12H, t, *J*= 6.83 Hz) (See Fig. S12). ^{13}C NMR ($CDCl_3$): 221.7 (***C***=S), 170.4 (***C***=O), 62.9 (***C***H$_2$-O), 47.3 (CH$_3$***C***H-S), 42.3 (***C***), 37.5 (-***C***H$_2$-S), 31.9 (-***C***H$_2$-CH$_2$-S), 29.7, 29.6, 29.5, 29.3, 29.1, 29 (-***C***H$_2$-CH$_2$-CH$_2$-S), 16.4 (***C***H$_3$CH-S), 14.1 (-***C***H$_3$) (See Fig. S13). R_f = 0.72 (hexanes:CH_2Cl_2 1:1).

Synthesis of macroCTA's (polymerization of PCAE using the CTA's 1 to 3 by RAFT)

Synthesis of macroCTA-**1** or $PCAE_3$-*b*-PEG_{45}-*b*-$PCAE_3$

CTA-**1** (162 mg, 0.06 mmol), CAE (0.45 g, 0.888 mmol) and AIBN (2 mg, 0.012 mmol) were dissolved in 2 mL of DMF. The ratio between CAE/CTA-1/AIBN =74/5/1. The solution was de-oxygenated by bubbling nitrogen for 20 min at room temperature. Then, the flask was placed in an oil bath preheated at 68 ^{o}C. After 5 h, the polymerization was stopped by cooling to room temperature. The polymerization yield was obtained gravimetrically by adding a three-fold excess of diethyl ether. The polymer was obtained as a yellowish thick liquid (65%); $M_{n\ NMR}$= [($I_{18'}$/3 (506.68 g/mol)/I_f2] + 2000 g/mol + 698 g/mol= 5,502 g/mol; DP_{PCAE}= 6; $M_{n,GPC}$=6,036 g/mol, Đ=1.10 (Table 1).

^{1}H NMR ($CDCl_3$, 400 MHz), δ (ppm): 4.86 (CH_3-C***H***-S, m, 2H), 4.28 (O-C$\boldsymbol{H}_2$-C$\boldsymbol{H}_2$-O, from PCAE), 3.98 (s, 12' C***H***), 3.84 (s, 7' C***H***), 3.65 (-C$\boldsymbol{H}_2$-C$\boldsymbol{H}_2$-O, s, 184H), 1.0 (s, 19'-C$\boldsymbol{H}_3$), 0.89 (s, 21'-C$\boldsymbol{H}_3$), 0.69 (s, 18'-C$\boldsymbol{H}_3$) (See Fig. S14).

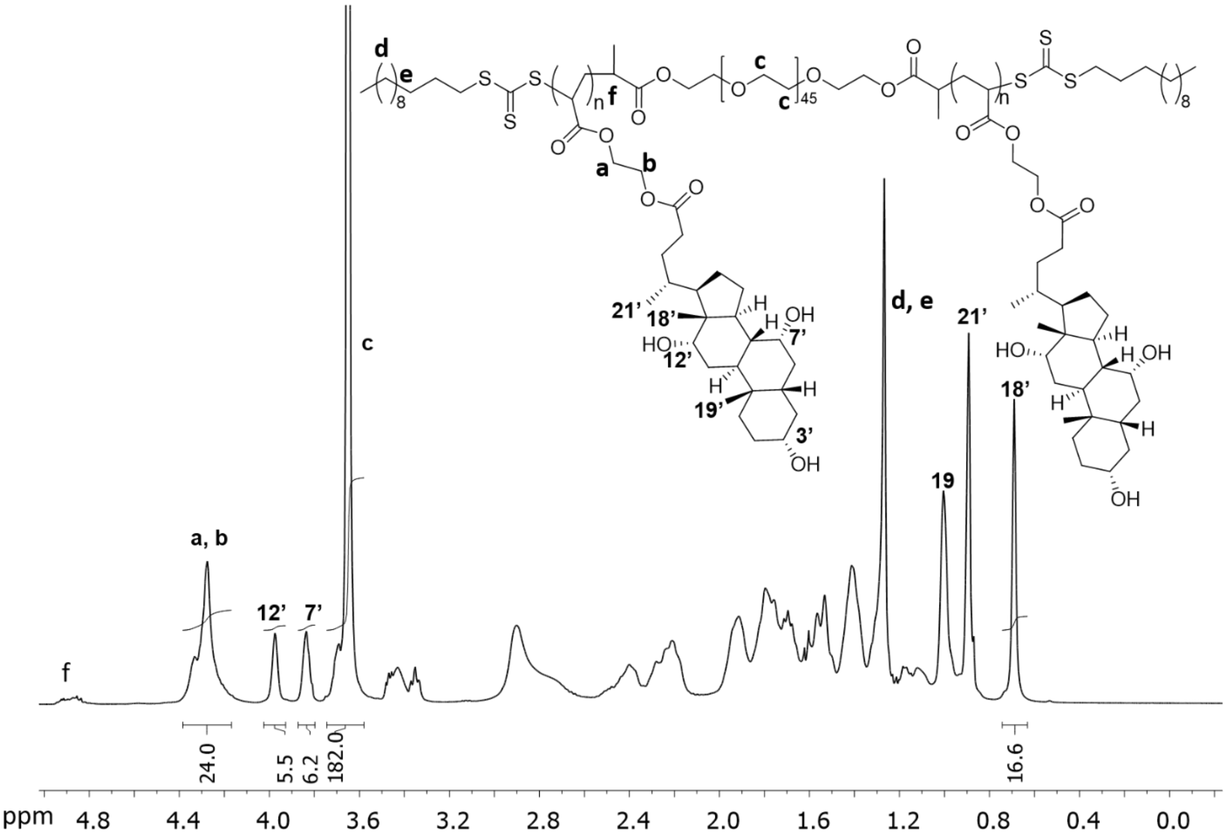

Figure S14. ^{1}H-NMR (400 MHz) spectrum of macroCTA-**1** in $CDCl_3$.

Synthesis of $(GE_7$-*b*-$PCAE_4)_3$ or macroCTA-**2**

CTA-**2** (370 mg, 0.181 mmol), CAE (1.3 g, 2.57 mmol) and AIBN (6 mg, 0.036 mmol) were dissolved in 4 mL of DMF. The ratio between CAE/CTA-**1**/AIBN = 71/5/1. The solution was de-oxygenated by bubbling nitrogen for 20 min at room temperature. Then, the flask was placed in an oil bath preheated at 68 ^oC. After 2 h, the polymerization was stopped by cooling to room temperature. The polymerization yield was obtained gravimetrically by adding a three-fold excess of diethyl ether. The polymer was obtained as a yellowish solid (60%); $M_{n\ NMR}$= [($I_{18'}$/3 (506.68 g/mol) /I_f/3] + 1,000 g/mol + 1,047 g/mol= 7,333 g/mol; DP_{PCAE}=12; $M_{n,GPC}$=8,519 g/mol, Đ=1.06 (Table 1).

^1H NMR ($CDCl_3$, 400 MHz), δ (ppm): 4.89 (CH_3-C***H***-S, m, 3H), 4.29 (O-C***H***$_2$-C***H***$_2$-O, from PCAE), 3.99 (s, 12' C***H***), 3.85 (s, 7' C***H***), 3.66 (-C***H***$_2$-C***H***$_2$-O, s, 87H), 1.01 (s, 19'-C***H***$_3$), 0.90 (s, 21'-C***H***$_3$), 0.70 (s, 18'-C***H***$_3$) (See Fig. S15).

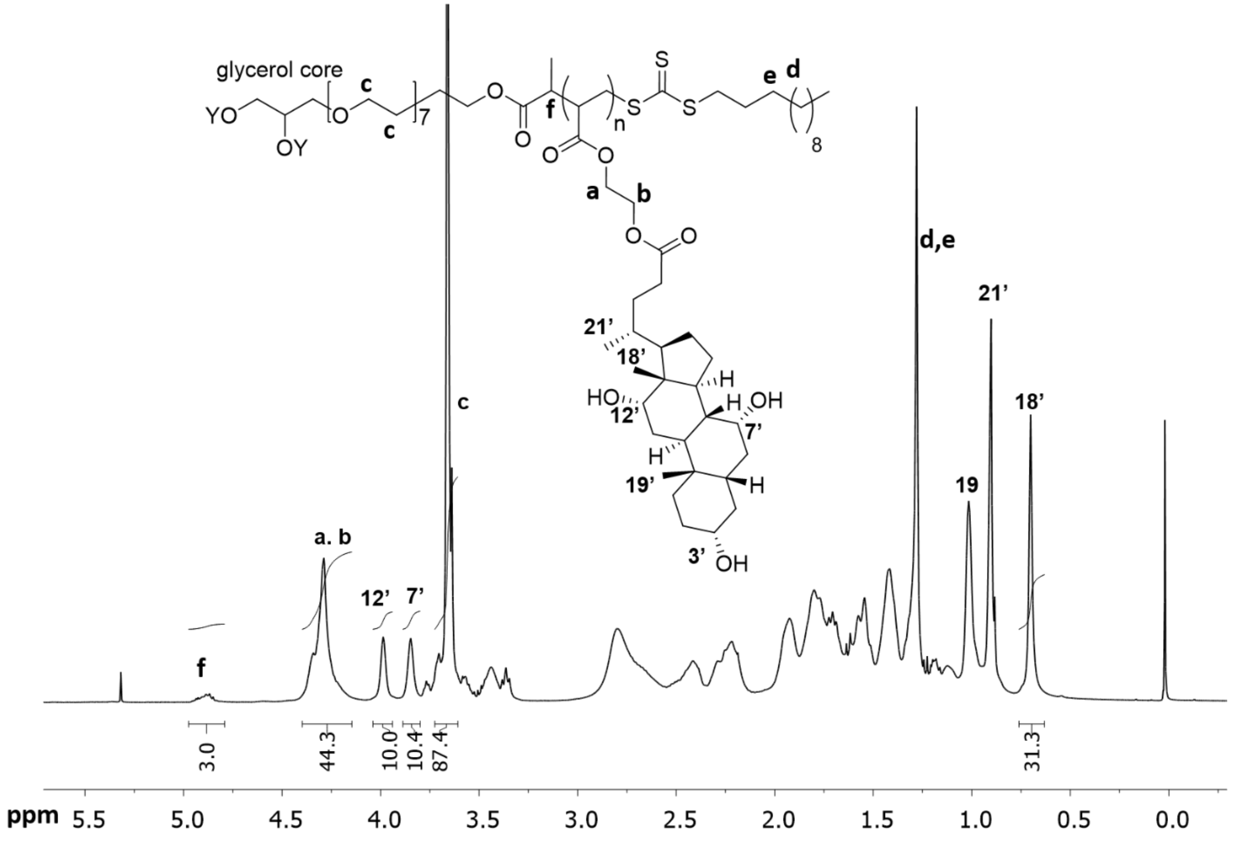

Figure S15. ^{1}H-NMR (400 MHz) spectrum of macroCTA-**2** in $CDCl_3$.

Synthesis of $(PCAE_2)_4$ or macroCTA-**3**

CTA-**3** (176 mg, 0.120 mmol), CAE (1.0 g, 1.97 mmol) and AIBN (4 mg, 0.024 mmol) were dispersed in 3.5 mL of DMF. The ratio between CAE/CTA-**3**/AIBN = 80/5/1. (Note: the CTA-**3** is only partially soluble in DMF, but at 68 ^oC become part of the solution in the first 3 min). The solution was de-oxygenated by bubbling nitrogen for 20 min at room

temperature. Then, the flask was placed in an oil bath preheated at 68 ^{o}C. After 1.5 h, the polymerization was stopped by cooling to room temperature. The polymerization yield was obtained gravimetrically by adding a three-fold excess of diethyl ether. The polymer was obtained as a yellowish solid (44%), $M_{n\ NMR}$= [($I_{18'}$/3 (506.68 g/mol)/I_f/4] + 1465 g/mol=5,079 g/mol; DP_{PCAE}= 2 per arm; $M_{n,GPC}$=7,200 g/mol, Đ=1.12 (Table 1).

MacroCTA-**3**. ^{1}H NMR ($CDCl_3$, 400 MHz), δ (ppm): 4.89 (CH_3-C***H***-S, m, 4H), 4.27 (O-C$\boldsymbol{H}_2$-C$\boldsymbol{H}_2$-O, from PCAE), 3.93 (s, 12’ C***H***), 3.86 (s, 7’ C***H***), 3.66 (-C$\boldsymbol{H}_2$-C$\boldsymbol{H}_2$-O, s, 87H), 1.0 (s, 19’-C$\boldsymbol{H}_3$), 0.89 (s, 21’-C$\boldsymbol{H}_3$), 0.69 (s, 18’-C$\boldsymbol{H}_3$) (See Fig. S16).

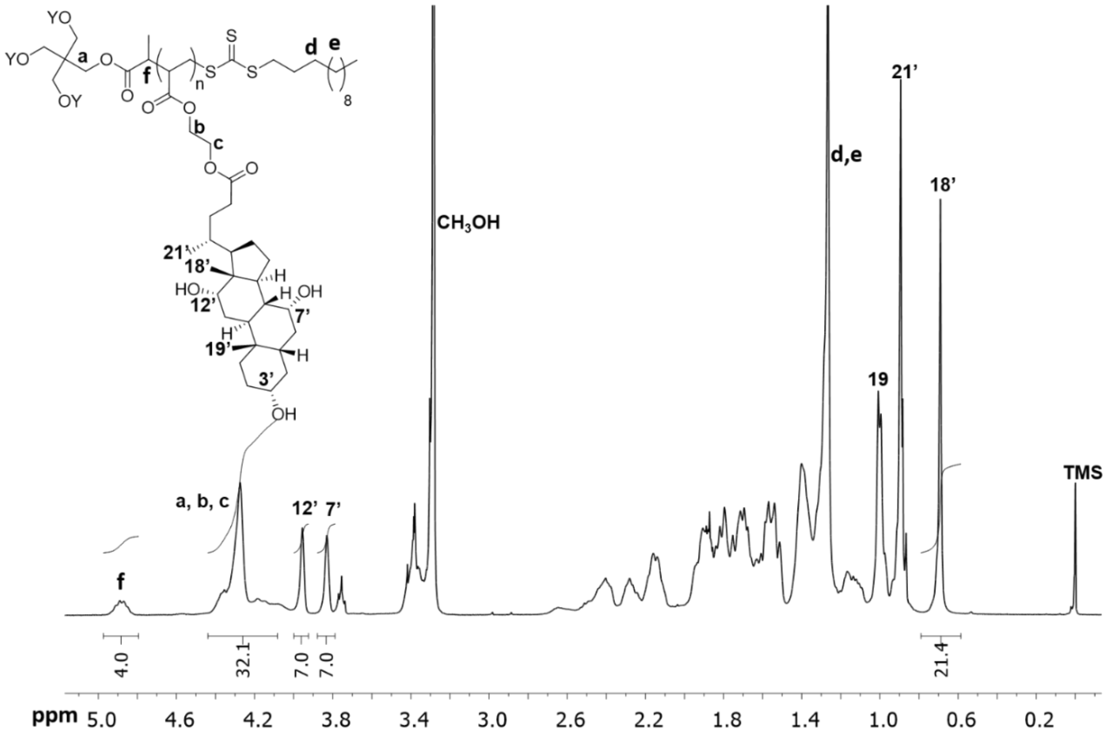

Figure S16. ^{1}H-NMR (400 MHz) spectrum of macroCTA-**3** in $CDCl_3/CD_3OD$.

Chain extension polymerization of NIPAM with or without acrylic acid in presence of the macroCTA's (Table 2)

As a representative example, the procedure is described for ($PCAE_2$-*b*-$PNIPAM_{93}$-*co*-$PAAc_{2\%}$)$_4$ copolymer (Table 2): macroCTA-**3** (0.064 g, 0.0126 mmol), NIPAM (0.49 g, 4.33 mmol), acrylic acid (0.006 g, 0.0884 mmol), and AIBN (0.36 mg, 0.0022 mmol) were dissolved in 2.5 mL of DMF. The oxygen was removed purged with nitrogen for 15 min. The mixture was heated in an oil bath at 68 ^{o}C for 6 h. The polymerization was obtained by adding a three-fold excess of diethyl ether. (PCAE-*b*-PNIPAM-*co*-$PAAc_{2\%}$)$_4$ copolymer product was obtained as a yellowish solid with 73% conv. Table 2, $M_{n,GPC}$=49,800 g/mol, Đ=1.28.

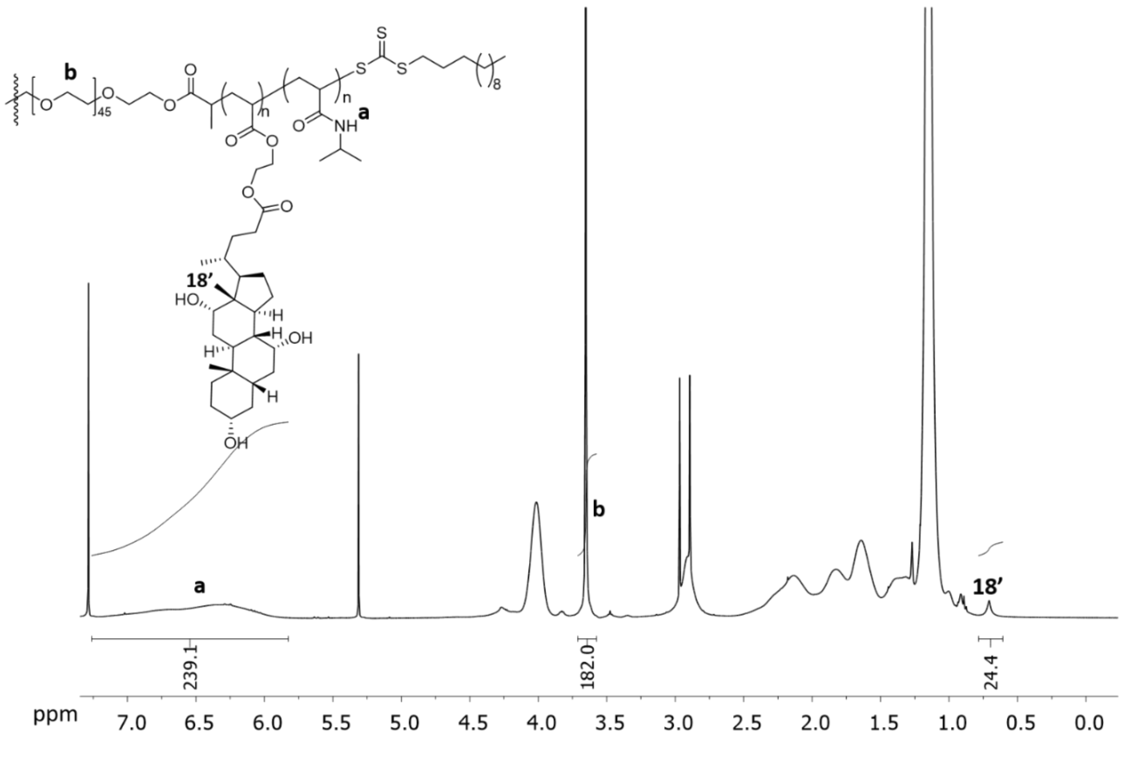

Figure S17. ^{1}H-NMR (400 MHz) spectrum of $PNIPAM_{120}$-*b*-$PCAE_3$-*b*-PEG_{45}-*b*-$PCAE_3$-*b*-$PNIPAM_{120}$ copolymer in $CDCl_3$.

^{1}H NMR ($CDCl_3$, 400 MHz), δ (ppm): 7.24-5.75 (-CO-N***H***-), 3.65 (-C***H***$_2$-C***H***$_2$-O, s), 0.70 (s, 18'-C***H***$_3$) (See Fig. S17).

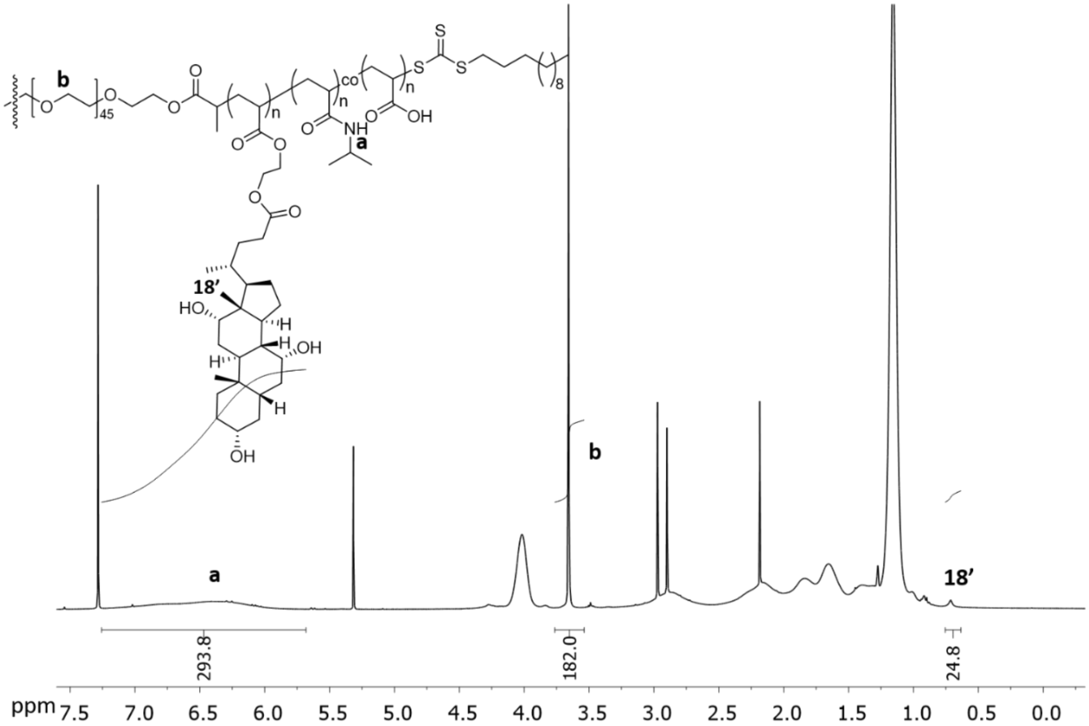

Figure S18. ^{1}H-NMR (400 MHz) spectrum of PAAc$_{2\%}$-*co*-PNIPAM$_{147}$-*b*-PCAE$_3$-*b*-PEG$_{45}$-*b*-PCAE$_3$-*b*-PNIPAM$_{147}$-*co*-PAAc$_{2\%}$ in $CDCl_3$.

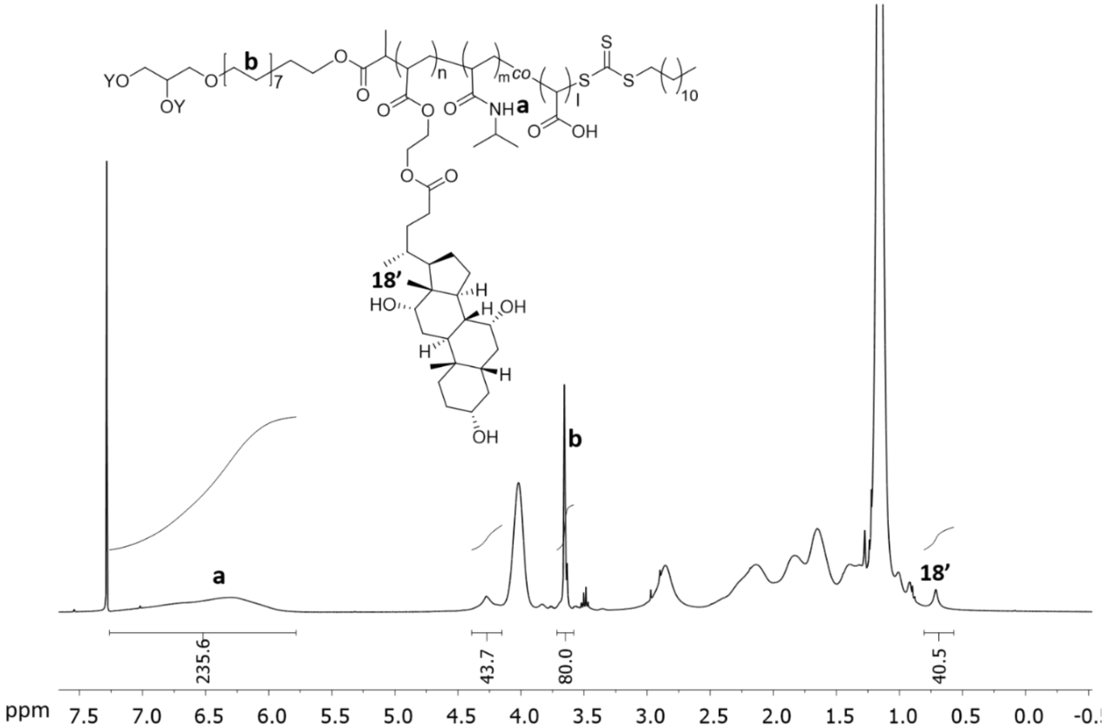

Figure S19. ^{1}H-NMR (400 MHz) spectrum of (GE$_7$-*b*-PCAE$_4$-*b*-PNIPAM$_{79}$)$_3$ in CDCl$_3$.

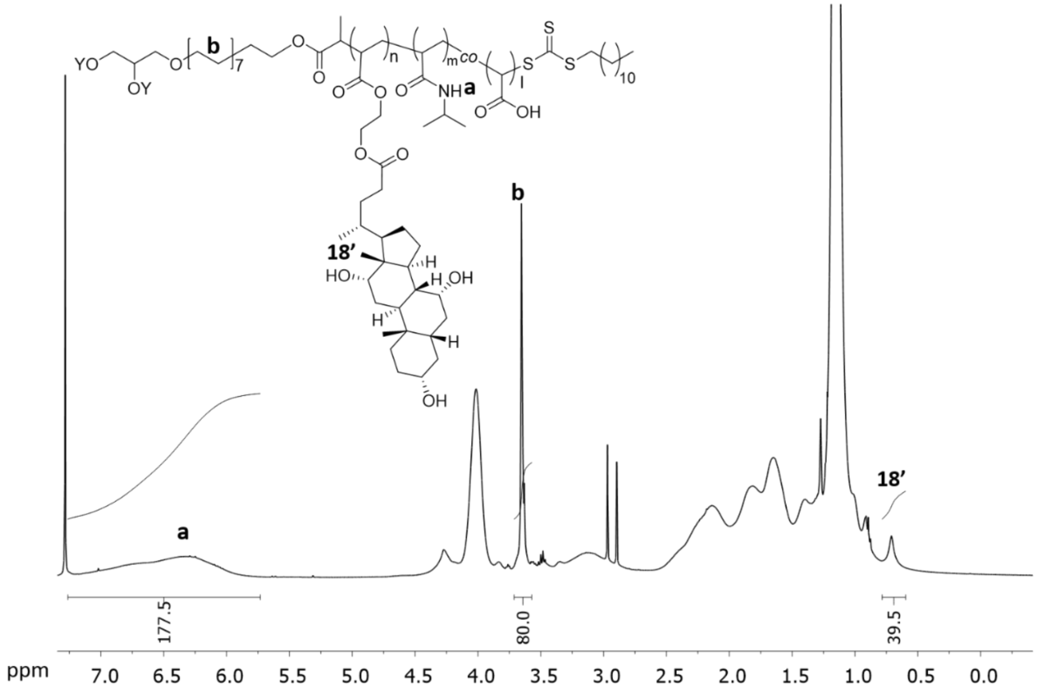

Figure S20. ^{1}H-NMR (400 MHz) spectrum of (GE$_7$-*b*-PCAE$_4$-*b*-PNIPAM$_{59}$-*co*-AAc$_{2\%}$)$_3$ in CDCl$_3$.

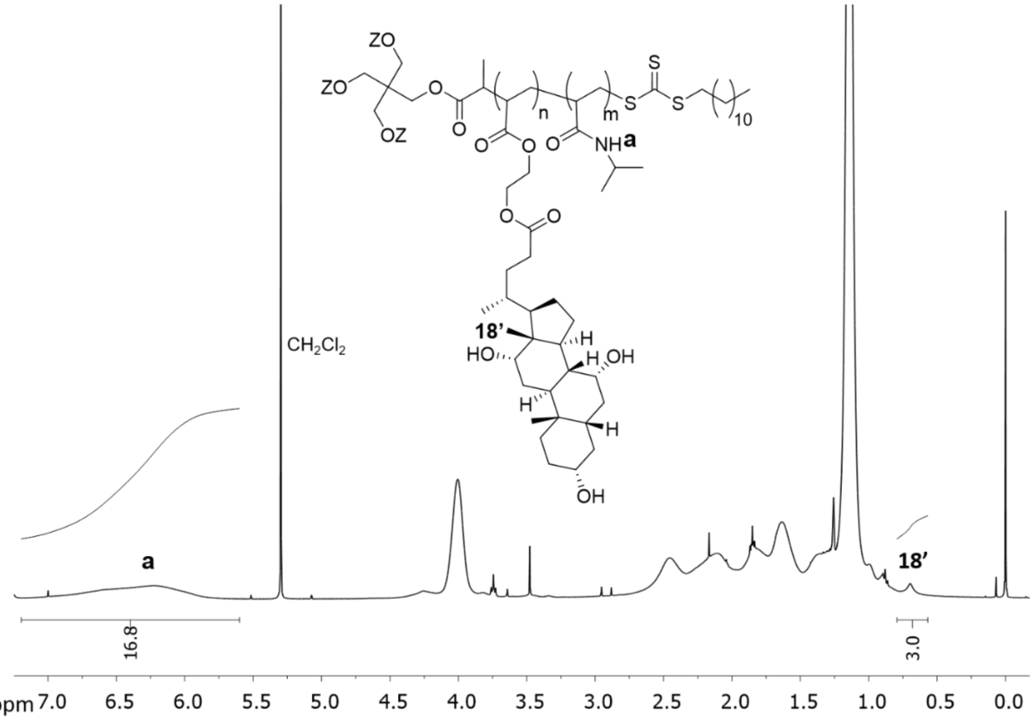

Figure S21. ^{1}H-NMR (400 MHz) spectrum of (PCAE$_2$-*b*-PNIPAM$_{75}$)$_4$ copolymer in $CDCl_3$.

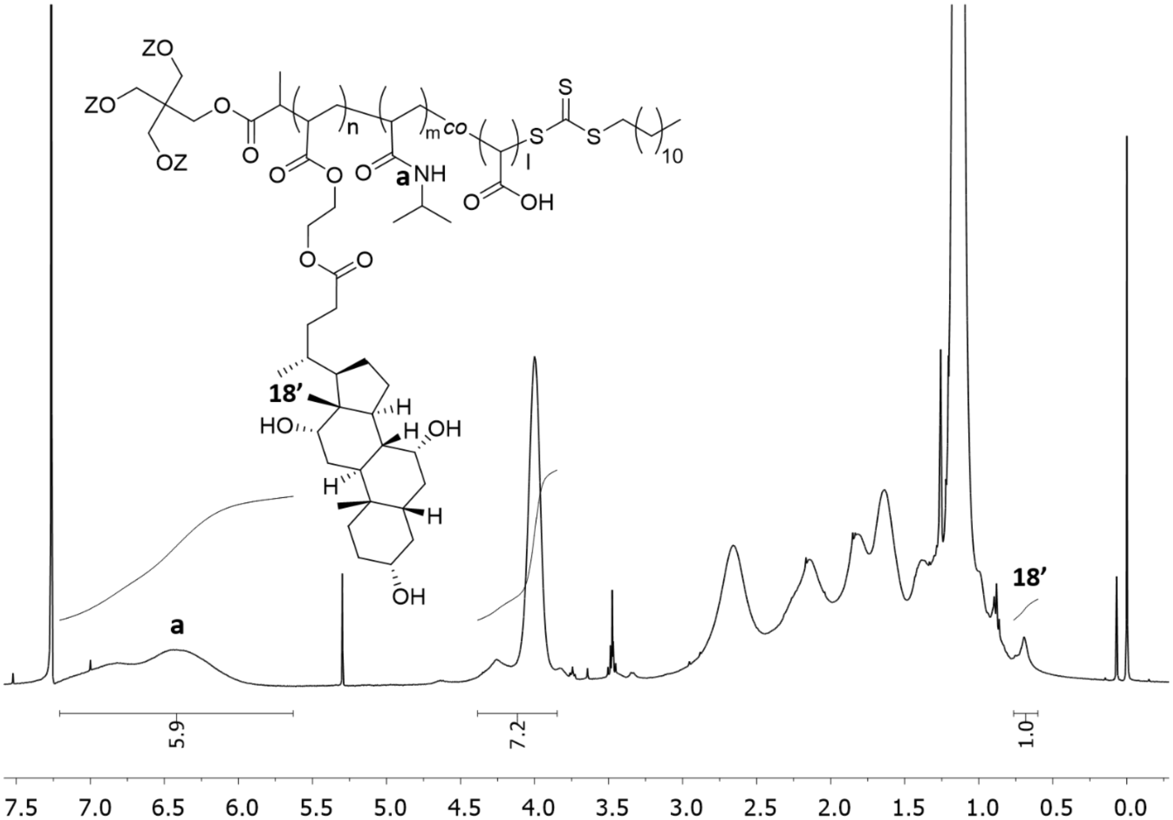

Figure S22. ^{1}H-NMR (400 MHz) spectrum of (PCAE$_2$-*b*-PNIPAM$_{93}$-*co*-AAc$_{2\%}$)$_4$ copolymer in $CDCl_3$.

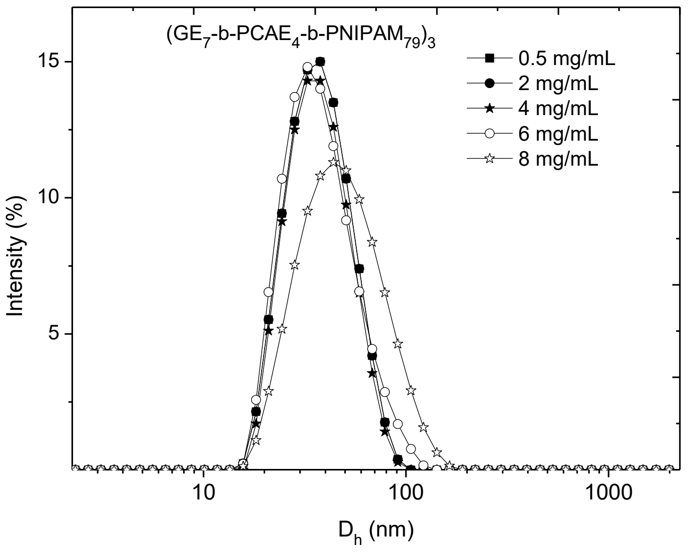

Figure S23. Hydrodynamic diameter (D_h) for (GE_7-*b*-$PCAE_4$-*b*-$PNIPAM_{79}$)$_3$ copolymer at different concentrations (0.5 to 8 mg/mL in PBS) by DLS at 25 ºC.

References:

[1] Pal, S.; Roy, G.;De, P. Polym. Chem. **2014**, *5*, 1275-1284.
